# A review of lifestyle, metabolic risk factors, and blood‐based biomarkers for early diagnosis of pancreatic ductal adenocarcinoma

**DOI:** 10.1111/jgh.14576

**Published:** 2019-01-17

**Authors:** Yuanjie Pang, Michael V Holmes, Zhengming Chen, Christiana Kartsonaki

**Affiliations:** ^1^ Clinical Trial Service Unit & Epidemiological Studies Unit, Nuffield Department of Population Health University of Oxford Oxford UK; ^2^ Medical Research Council Population Health Research Unit, Nuffield Department of Population Health University of Oxford Oxford UK; ^3^ National Institute for Health Research Oxford Biomedical Research Centre Oxford University Hospital Oxford UK

**Keywords:** biomarkers, early diagnosis, metabolomics, pancreatic ductal adenocarcinoma, proteomics, risk factors

## Abstract

We aimed to review the epidemiologic literature examining lifestyle and metabolic risk factors, and blood‐based biomarkers including multi‐omics (genomics, proteomics, and metabolomics) and to discuss how these predictive markers can inform early diagnosis of pancreatic ductal adenocarcinoma (PDAC). A search of the PubMed database was conducted in June 2018 to review epidemiologic studies of (i) lifestyle and metabolic risk factors for PDAC, genome‐wide association studies, and risk prediction models incorporating these factors and (ii) blood‐based biomarkers for PDAC (conventional diagnostic markers, metabolomics, and proteomics). Prospective cohort studies have reported at least 20 possible risk factors for PDAC, including smoking, heavy alcohol drinking, adiposity, diabetes, and pancreatitis, but the relative risks and population attributable fractions of individual risk factors are small (mostly < 10%). High‐throughput technologies have continued to yield promising genetic, metabolic, and protein biomarkers in addition to conventional biomarkers such as carbohydrate antigen 19‐9. Nonetheless, most studies have utilized a hospital‐based case–control design, and the diagnostic accuracy is low in studies that collected pre‐diagnostic samples. Risk prediction models incorporating lifestyle and metabolic factors as well as other clinical parameters have shown good discrimination and calibration. Combination of traditional risk factors, genomics, and blood‐based biomarkers can help identify high‐risk populations and inform clinical decisions. Multi‐omics investigations can provide valuable insights into disease etiology, but prospective cohort studies that collect pre‐diagnostic samples and validation in independent studies are warranted.

## Introduction

Pancreatic ductal adenocarcinoma (PDAC) has the highest case fatality rate of all cancers.^1^, ^2^, ^3^ It has a median survival of 4 to 6 months and a 5‐year survival of less than 5%.^1^, ^2^ More than 80% of PDAC patients are diagnosed at a late stage (stages III and IV), and 20–25% of the patients have localized, surgically resectable tumors.^4^ This is because of the unspecific and late‐presenting signs and symptoms of PDAC (e.g. nausea and vomiting, bloating, abdominal pain, weight loss, jaundice, and newly onset diabetes) and the inaccessible location of the pancreas.^1^, ^2^ Despite its dismal prognosis, survival of PDAC is higher when it is diagnosed at an early stage. Compared with the overall 5‐year survival of less than 5%, Cancer Research UK data showed a 5‐year survival of 7–25% for resectable PDAC.^5^ Similarly, Surveillance, Epidemiology, and End Results data in the USA (2006–2012) showed a 5‐year survival of ~30% for localized PDAC, 11% for regional lymph node spread tumor, and ~3% for distant metastasis.^6^ Furthermore, recent genome sequencing data suggested that it takes at least 10 years between the initiating mutation and the birth of the parental founder cell and an additional 5 years between the tumor initiation and the acquisition of metastatic ability.^7^ These data demonstrate a potential window of opportunity for early detection of PDAC if diagnostic biomarkers were available.

**Figure 1:**
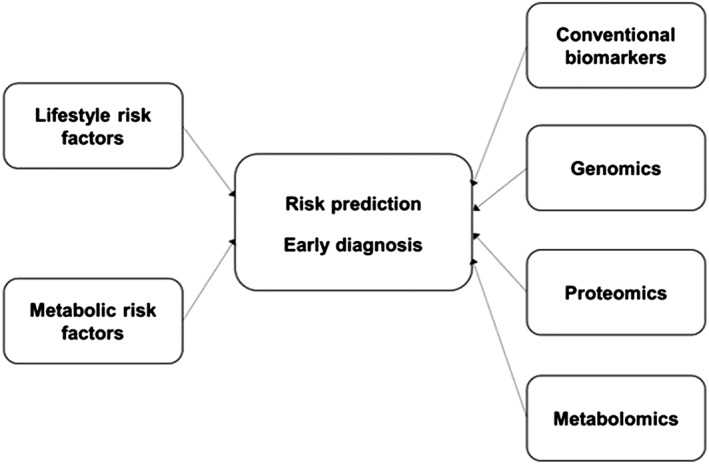
Steps towards early diagnosis of pancreatic ductal adenocarcinoma. Risk prediction models that are currently available include socio‐demographics, lifestyle risk factors, medical history, and, for some, genetic variants. Ideally, biomarkers can be incorporated into these models. The current recommendation is selective screening of individuals at increased risk for PDAC based on their family history or identifiable genetic predisposition.^73^ The current screening modalities include endoscopic ultrasonography and/or magnetic resonance imaging/magnetic resonance cholangiopancreatography but not biomarkers.^73^ Lifestyle risk factors including smoking, alcohol, and diet are behavioral factors that are potentially modifiable. Metabolic risk factors, especially those related to the insulin resistance syndrome, are important risk factors for PDAC. These include adiposity, diabetes, hyperglycemia, physical activity, and metabolic syndrome. Other possible risk factors for PDAC are reviewed elsewhere.^11^ CA 19‐9 is the only conventional biomarker that has been demonstrated to be clinically useful, despite its relatively low sensitivity and specificity. Genomic investigations of PDAC have identified genetic syndromes or mutations in familial PDAC and genetic polymorphisms in sporadic PDAC. Proteomics is the comprehensive characterization of the identity, characteristics, and interactions of the proteins found in individual cellular systems.^40^ Metabolomics is the comprehensive characterization of small low‐molecular‐weight metabolites in biological samples.^41^ Both metabolomics and proteomics can provide coverage of metabolites and proteins in much greater quantities than traditional laboratory approaches.

Prospective studies so far have reported over 20 potential risk factors for PDAC, primarily lifestyle and metabolic risk factors, while case–control studies have suggested the clinical utility of genomics, proteomics, and metabolomics assays in early diagnosis of PDAC. An integrated approach of traditional risk factors and biomarkers may improve our understanding of risk prediction and early diagnosis of PDAC. This review gives a timely overview in these areas and can be particularly helpful to inform large‐scale population‐based studies given the readily available resources. In this article, we will review (i) epidemiologic studies of lifestyle and metabolic risk factors for PDAC (mainly smoking, alcohol, diet, adiposity, diabetes, and physical activity), genome‐wide association studies (GWAS), and risk prediction models incorporating these factors and (ii) epidemiologic studies of blood‐based biomarkers for PDAC (conventional diagnostic markers, metabolomics, and proteomics). As summarized in Figure 1, these factors have the potential to be predictive of PDAC risk and can provide valuable insights into risk prediction, early diagnosis, and treatment of PDAC. Familial PDAC with identified genetic susceptibility will not be discussed in detail. Other potential biomarkers including cell‐free noncoding RNA, circulating tumor DNA (ctDNA), circulating tumor cells, exosomes, and the microbiome have been reviewed elsewhere and will not be discussed in detail (Table S1).^8^, ^9^, ^10^

## Lifestyle and metabolic risk factors

Pancreatic ductal adenocarcinoma has a multifactorial etiology, and several risk factors have been reported.^11^ Table 1 shows the study information and pooled estimates from published meta‐analyses of prospective studies. Despite the large number suggested, the majority of risk factors have a relative risk (RR) of less than 2, and if causal associations are assumed, the population attributable fraction (PAF) of individual risk factors is small.^11^ For example, the PAF for smoking ranges from 11% to 32%, while PAFs for other risk factors are less than 10%.^11^ Future studies assessing the combined effects of risk factors may inform risk prediction of PDAC and help identify at‐risk populations. Furthermore, an appreciable proportion of PDAC cases cannot be explained by established risk factors, and therefore, other risk factors need to be investigated, such as infections, medications, and immunity.

**Table 1:** Associations of lifestyle and metabolic risk factors with PDAC from systematic reviews and meta‐analyses

Reference	Year	Risk factor	Category	No. of studies	No. of cases	Result	*I* ^2^ (%)
Iodice *et al*.^12^	2008	Smoking	Former *vs* never	19	—	1.21 (1.10, 1.35)	—
			Current *vs* never	26	—	1.70 (1.53, 1.90)	—
Tramacere *et al*.^13^	2010	Alcohol	< 3 drinks per day†	7	4384	0.12 (0.86, 0.95)	—
			≥ 3 drinks per day†	5	797	1.30 (1.16, 1.47)	—
World Cancer Research Fund^14^	2011	Alcohol	Per 10 g/day	9	3096	1.00 (0.99, 1.01)	0
			High *vs* low	9	3096	1.30 (1.09, 1.54)	0
World Cancer Research Fund^14^	2011	Fruit	Per 100 g/day	5	1532	1.00 (0.95, 1.05)	0
World Cancer Research Fund^14^	2011	Red meat	100 *vs* 20 g/day	8	2761	1.19 (0.98, 1.45)	52
World Cancer Research Fund^14^	2011	Processed meat	50 *vs* 20 g/day	7	2748	1.17 (1.01, 1.34)	0
World Cancer Research Fund^14^	2011	Fish	Per 20 g/day	7	3372	1.03 (0.97, 1.08)	0
World Cancer Research Fund^14^	2011	Coffee	Per cup per day	13	1460	1.02 (0.95, 1.09)	29
World Cancer Research Fund^14^	2011	Saturated fatty acids	Per 10 g/day	5	2740	1.11 (1.01, 1.21)	43
World Cancer Research Fund^14^	2011	Fructose	Per 25 g/day	6	2831	1.22 (1.08, 1.37)	0
World Cancer Research Fund^14^	2011	Total physical activity	Per 20 MET‐h/day	3	687	0.81 (0.64, 1.02)	0
		Leisure‐time physical activity	Per 10 MET‐h/day	5	1315	0.99 (0.96, 1.03)	0
Behrens *et al*.^15^	2015	Total physical activity	High *vs* low	5	1037	0.91 (0.69, 1.19)	—
		Leisure‐time physical activity	High *vs* low	18	6461	0.95 (0.90, 1.01)	—
Aune *et al*.^16^	2012	BMI	Per 5 kg/m^2^	23	9504	1.10 (1.07, 1.14)	19
		WC	Per 10 cm	5	949	1.11 (1.05, 1.18)	0
		WHR	Per 0.1	4	1047	1.19 (1.09, 1.31)	11
Pang *et al*.^17^	2017	Young adult BMI	Per 5 kg/m^2^	5	4602	1.18 (1.12, 1.24)	84
World Cancer Research Fund^14^	2011	Height	Per 5 cm	14	6147	1.07 (1.03, 1.12)	57
Pang *et al*.^18^	2017	Diabetes	Yes *vs* no	22	14 211	1.52 (1.43, 1.63)	55
Pang *et al*.^18^	2017	Fasting blood glucose	Per 1 mmol/L	1	139	1.11 (1.02, 1.20)	—
		Random blood glucose	Per 1 mmol/L	3	1451	1.15 (1.09, 1.21)	70
		Post‐load blood glucose	Per 1 mmol/L	4	576	1.13 (1.08, 1.19)	54
Duell *et al*.^19^	2012	Pancreatitis (> 2 years)	Yes *vs* no	10	5048	2.71 (1.96, 3.74)	—

† One drink per day = 12.5 g ethanol. Reference category: nondrinkers and occasional drinkers (< 0.5 drinks per day).

BMI, body mass index; MET, metabolic equivalent of task; PDAC, pancreatic ductal adenocarcinoma; WC, waist circumference; WHR, waist‐to‐hip ratio.

### Lifestyle risk factors

Lifestyle risk factors including smoking, alcohol drinking, and diet have been investigated in relation to risk of PDAC. Among these lifestyle factors, smoking is the most well‐established one. A meta‐analysis of 35 prospective cohort studies with 14 236 PDAC cases reported a 70% and 20% excess risk among current and former smokers, respectively.^12^ Among current smokers, there were also moderate dose–response relationships with amount and duration smoked, with each 20 cigarettes per day and each 10‐year smoking duration associated with 60% and 16% higher risk, respectively.^12^ Heavy alcohol drinking is also associated with higher risk of PDAC, while the effects of light‐to‐moderate drinking remain unclear. Previous prospective studies have shown that heavy alcohol drinking (i.e. ≥ 3 drinks or 36 g alcohol per day) is associated with a 30% higher risk of PDAC, whereas light‐to‐moderate drinking is not associated.^13^ Although the role of diet in relation to PDAC risk has been inconclusive, prospective studies have suggested that low consumption of red meat and processed meat and high consumption of fresh fruits are associated with lower risk. A meta‐analysis of eight prospective cohort studies involving 2761 PDAC cases reported an RR of 1.19 (0.98–1.45) comparing 100 *versus* 20 g/day of red meat intake,^14^ while another meta‐analysis of seven prospective cohort studies involving 2748 PDAC cases reported an RR of 1.17 (1.01–1.34) comparing 50 *versus* 20 g/day of processed meat intake.^14^ A meta‐analysis of five prospective cohort studies involving 1532 PDAC cases reported a null association between fruit intake and PDAC risk (RR 1.00, 0.95–1.05, per 100 g/day).^14^

### Metabolic risk factors

In addition to lifestyle factors, metabolic risk factors that are related to the insulin resistance syndrome may play a role in the etiology of PDAC. Physical activity is associated with improved insulin sensitivity, lower blood glucose, and lower risk of developing type 2 diabetes.^14^ However, previous prospective studies have been inconclusive whether physical activity is associated with risk of PDAC. In the meta‐analysis conducted by the World Cancer Research Fund (WCRF), each 20 metabolic equivalent of task‐hours per day (MET‐h/day) higher total physical activity was associated with ~20% nonsignificantly lower risk of PDAC (RR per 20 MET‐h/day 0.81 [0.64–1.02]), while leisure‐time physical activity was not related to PDAC (RR per 10 MET‐h/day 0.99 [0.96–1.03]).^14^ However, this meta‐analysis included a limited number of PDAC cases, with 687 cases for total and 1315 cases for leisure‐time physical activity. Similar to the WCRF systematic literature review, a recent meta‐analysis of prospective studies showed that neither total physical activity nor leisure‐time physical activity was associated with risk of PDAC, despite a greater number of PDAC cases that were included (*n* = 8353).^15^ The summary of RR comparing high *versus* low categories was 0.91 (0.69–1.19) for total and 0.95 (0.90–1.01) for leisure‐time physical activity.^15^

Adiposity is an established risk factor for PDAC, and the WCRF has judged that evidence that body fatness is a cause of PDAC is convincing.^14^ General adiposity, as assessed by body mass index (BMI) measured or self‐reported in middle‐to‐old ages, is positively associated with risk of PDAC. A meta‐analysis involving 23 prospective cohort studies and 9504 PDAC cases reported a 10% higher risk associated with 5 kg/m^2^ higher adulthood BMI (RR 1.10, 1.07–1.14).^16^ Despite the small number of prospective studies, central adiposity (waist circumference or waist‐to‐hip ratio) is also positively associated with risk of PDAC. The same meta‐analysis reported an 11% higher risk associated with 10‐cm higher waist circumference (RR 1.11, 1.05–1.18, 5 studies, 949 PDAC cases) and a 19% higher risk associated with 0.1‐unit higher waist‐to‐hip ratio (RR 1.19, 1.09–1.31, 4 studies, 1047 PDAC cases). On the other hand, young adulthood adiposity, as assessed by self‐reported BMI at age 18–25 years, also shows a positive association with risk of PDAC.^17^ A recent meta‐analysis involving five prospective cohort studies and 4602 PDAC cases reported an 18% higher risk associated with 5 kg/m^2^ higher young adulthood BMI (RR 1.18, 1.12–1.24).^17^ PDAC has a long subclinical period in which unintentional weight loss might occur, and therefore, the association between adiposity and PDAC risk may be affected by reverse causation. Nonetheless, recent evidence from a Mendelian randomization study has shown that genetically higher BMI is associated with increased risk of PDAC (odds ratio 1.66 [1.05–2.63] per 4.6 kg/m^2^ higher BMI),^20^ suggesting a causal role of BMI in PDAC etiology.

Diabetes is associated with a 1.5‐fold to 2.5‐fold higher risk of PDAC.^11^ A recent meta‐analysis involving 34 prospective studies and 35 761 PDAC cases showed that participants with diabetes have a twofold higher risk (RR = 1.98 [1.92–2.03]), and the pooled RR was 1.52 (1.43–1.63) when restricting to 22 prospective cohort studies.^18^ The association of diabetes with PDAC is independent of obesity. A meta‐analysis of nine prospective studies reported a RR of 1.46 (1.36–1.56) when further adjusting for BMI.^18^ Among participants without diabetes, there is a positive association between blood glucose and risk of PDAC, with RRs of 1.11 (1.02–1.20), 1.15 (1.09–1.21), and 1.13 (1.08–1.19) per 1 mmol/L higher fasting blood glucose, random blood glucose, and post‐load blood glucose, respectively.^18^ The association of diabetes may also be confounded by reverse causation (i.e. diabetes may be a consequence rather than a cause of PDAC). Preclinical PDAC can induce diabetes due to beta‐cell dysfunction and insulin resistance.^21^ It has been estimated that approximately 40–50% of newly diagnosed PDAC patients have diabetes at diagnosis.^21^, ^22^, ^23^ Although hyperglycemia and hyperinsulinemia have been proposed as the underlying mechanisms linking diabetes and PDAC risk, a recent Mendelian randomization study reported no evidence of a causal relationship between type 2 diabetes and PDAC risk,^20^ suggesting that the positive associations in observational studies may be partly explained by reverse causality. However, it should be noted that Mendelian randomization studies rely on important assumptions.^24^ On the other hand, this study suggested that genetically increased plasma insulin (i.e. higher levels of plasma insulin predicted by genetic variants) was causally associated with PDAC risk, in line with previous observational evidence showing a positive association between plasma insulin and risk of PDAC. Indeed, prospective studies have suggested positive associations of plasma insulin, insulin‐like growth factors (IGFs), and IGF‐binding proteins (IGFBPs) with risk of PDAC, although the evidence has been inconclusive.^25^, ^26^, ^27^, ^28^, ^29^, ^30^, ^31^

Pancreatitis, an inflammatory disease of the pancreas, is also associated with risk of PDAC.^2^ Although alcohol, gallstones, and autoimmune diseases are the main causes of pancreatitis, metabolic risk factors including adiposity and diabetes are important risk factors for pancreatitis.^2^ Previous prospective studies have identified metabolic risk factors for pancreatitis including adiposity, hyperglycemia, and diabetes that are also risk factors for PDAC.^2^ Previous prospective and case–control studies have shown a higher risk of PDAC associated with a diagnosis of pancreatitis, with reported RR or odds ratio ranging from 2.7 to 13.^11^ Although the strong positive association may be partly due to reverse causation (i.e. pancreatic tumor‐related ductal obstruction) and misdiagnosis of PDAC as pancreatitis,^19^ previous studies showed higher risk of PDAC when excluding pancreatitis cases diagnosed within 2 years of PDAC diagnosis.^19^ Furthermore, a few small case–control studies have shown overexpression of biomarkers in both pancreatitis and PDAC (e.g. ephrin receptor A3 and fibrillin 1),^3^ while recent case–control studies that compared metabolomics and proteomics profiles of pancreatitis and PDAC can inform the shared etiology and differential diagnosis between the two diseases (see the sections on Metabolomics and Proteomics). More importantly, pancreatitis is predictive of subsequent PDAC diagnosis (see the section on Risk Prediction).

Apart from these lifestyle and metabolic risk factors mentioned earlier, other risk factors for PDAC have been reported, including hepatitis B virus (RR 1.2–1.4) and non‐O blood group (RR 1.3–1.4). Although the evidence has been inconclusive, history of allergy is associated with lower risk of PDAC, while regular use of aspirin and nonsteroidal anti‐inflammatory drugs is not associated with risk of PDAC.^11^ In addition, *Helicobacter pylori* infection and periodontitis are associated with higher risks of PDAC,^32^ possibly because of the increased inflammatory response and the interaction between the human microbiome and the immune system.^10^ However, the reported associations of *H. pylori* with PDAC risk have not been consistent.^11^ Although inflammation has been implicated in the etiology of PDAC, some prospective studies have shown null associations of inflammation markers with risk of PDAC, including interleukin (IL)‐6, C‐reactive protein, and tumor necrosis factor‐α.^33^, ^34^, ^35^

## Genomics

While Mendelian randomization studies utilize genetic instruments for putative risk factors to assess the causality of metabolic risk factors in relation to the disease, large‐scale, trans‐ethnic GWAS have continued to identify common genetic variants that influence disease risk. For sporadic PDAC, previous GWAS have identified at least 22 common variants in primarily European populations (Table S2). Recent GWAS have identified five new susceptibility loci among participants of Chinese descent and have suggested three loci among participants of Japanese descent.^36^, ^37^ Despite the larger number of variants/loci identified compared with familial PDAC, the RRs are relatively small, mostly ranging from 0.7 to 1.3. Although GWAS findings advance our understanding of the development of PDAC, future studies are warranted to investigate the biological mechanisms of these common susceptibility alleles and to incorporate the genetic information to develop risk prediction models. Furthermore, future genetic risk models need to incorporate genetic variants of a wide range of allele frequencies, including rare, low‐frequency, and common variants.^38^

On the other hand, the genetic basis for familial PDAC remains poorly understood, although several genetic factors for PDAC have been identified. A positive family history of PDAC has been reported to be associated with an 80–200% higher risk.^11^ Previous reviews have summarized inherited disorders that carry an increased risk of PDAC, the genes involved, and the corresponding RRs.^1^, ^2^, ^8^ These involved germline mutations in BRCA1, BRCA2, CDK2A, STK11, PRSS1, SPINK1, PALB2, ATM, and CFTR, and the associated RRs ranged from 2.2 to over 100. However, these genetic alterations are rare in the general population and only account for approximately 10% of all PDAC cases.^2^

## Blood‐based biomarkers

Blood sampling through venesection is a noninvasive and cost‐effective approach that can provide high‐throughput diagnostic information.^2^ Investigating blood‐based biomarkers can provide insights into the biological mechanisms. Conventional assays including traditional tumor biomarkers (e.g. carbohydrate antigen 19‐9 [CA 19‐9], carcinoembryonic antigen [CEA], and carbohydrate antigen 125 [CA‐125]) are readily available in clinical settings. In addition to traditional tumor biomarkers, proteomics and metabolomics (including lipidomics) have recently become more feasible allowing the identification of promising clinical biomarkers.^39^, ^40^, ^41^, ^42^ An exhaustive examination of potential biomarkers for PDAC is beyond the scope of this review. A compendium of 441 secreted proteins overexpressed in pancreatic cancer has been reported elsewhere.^3^

### Conventional biomarkers

Carbohydrate antigen 19‐9 (CA 19‐9) is a Lewis antigen of the mucin 1 protein class, a well‐established blood test for the early detection of PDAC.^2^ As the most extensively evaluated marker for PDAC, CA 19‐9 has poor specificity for PDAC, and the use of CA 19‐9 alone for PDAC screening has been discouraged.^43^ Although CA 19‐9 has been reported to discriminate between symptomatic individuals and healthy controls (sensitivity 80%, 95% confidence interval [CI] 78–83%; specificity 80%, 95% CI 78–82%)^2^ and benign pancreatic disease (sensitivity 78%, 95% CI 72–80%; specificity 83%),^44^ it has been shown to be ineffective in the mass screening of asymptomatic subjects.^45^ Another limitation is that CA 19‐9 is elevated in patients with nonmalignant diseases, including liver cirrhosis, chronic pancreatitis (CP), cholangitis, and other cancers of the gastrointestinal system.^46^ Moreover, CA 19‐9 is not expressed in Lewis blood‐type‐negative patients (approximately 5–10% of the population).^46^ Previous studies on CA 19‐9 collected blood samples after a diagnosis was made, whereas a recent study showed good diagnostic performance using pre‐diagnostic blood samples. This nested case–control study within the UK Collaborative Trial of Ovarian Cancer Screening with 154 cases collected samples taken up to 6 years before clinical presentation of PDAC.^47^ This study showed that at 95% specificity, CA 19‐9 (> 37 U/mL) had a sensitivity of 68% up to 1 year and 53% up to 2 years before diagnosis. This suggests that more than half of PDAC cases can be detected 1–2 years before clinical presentation. In addition, they showed that the combination of CA 19‐9 and CA‐125 improved sensitivity because CA‐125 was elevated in 20% of CA 19‐9‐negative cases.

Apart from CA 19‐9, alternative biomarkers have been investigated in early detection of PDAC, including tumor markers (e.g. CA‐125, CA‐242, α‐fetoprotein, and CEA), cytokines/chemokines (IL‐2, IL‐10, IL‐13, and tumor necrosis factor‐α), cell adhesion molecules (e.g. intercellular adhesion molecule 1), proteases/inhibitors in extracellular matrix degradation (e.g. matrix metalloproteinase and tissue inhibitor of metalloproteinase), acute‐phase reactants (e.g. C‐reactive protein and serum amyloid A), and other biomarkers (e.g. osteoprotegerin and IGF‐binding proteins [IGFBP2 and IGFBP3]).^46^, ^48^, ^49^ However, when combined with CA 19‐9, the majority of these biomarkers have not been shown to improve the diagnostic accuracy compared with CA 19‐9 alone.^46^ Although additional studies are needed to validate the use of these biomarkers, a few studies have reported that macrophage colony‐stimulating factor 1, haptoglobin, tumor‐specific growth factor, heat shock protein 27, clivatuzumab, mucin 1, CEA‐related cell adhesion molecule 1, mucin 5AC, and miR‐1290 had higher sensitivity and specificity for diagnosis of PDAC than CA 19‐9 alone.^3^, ^9^, ^50^

### Proteomics

In recent years, advances have been made in proteomics assays (mainly antibody microarrays) to capture the systemic immune response to cancer.^42^ As a consequence, multiplexed proteomics panels have been investigated with the aim of increasing sensitivity and specificity. Such a multiplexed serum biomarker signature has the potential to improve diagnosis accuracy of PDAC and to distinguish PDAC from benign conditions in case–control studies (number of PDAC cases 13–401, median 80; Tables 2,S3).^42^ However, there are several limitations for investigations of both proteomics and metabolomics: (i) the majority of case–control studies recruited PDAC cases of early and advanced stages and therefore could not distinguish between markers only present in advanced disease and biomarkers useful for early diagnosis, (ii) some of the results have not been validated in independent samples, and (iii) the diagnostic accuracy of proteomic assays was low in studies that collected pre‐diagnostic samples (number of PDAC cases, 87–174; Table 3), demonstrating the challenge for early diagnosis.

**Table 2:** Study information of case–control studies of proteomics and PDAC

Reference	No. of cases	Platform	Identified biomarker	Diagnostic performance
Wingren *et al*./2012/Sweden	34	Recombinant antibody microarray platform	A 25‐serum biomarker signature discriminating PDAC from the combined group of HC, CP, and AIP was determined	AUC: PDAC *vs* HC 0.95, PDAC *vs* CP 0.86, and PDAC *vs* AIP 0.99
Faca *et al*./2008/USA	13	Proteomic approach based on extensive protein fractionation	5 proteins that were upregulated in mouse plasma at the PanIN stage (LCN2, REG1A, REG3, TIMP1, and IGFBP4) together with CA 19.9	AUC: 5 proteins 0.817 and 5 proteins + CA 19‐9 0.911
Ingvarsson *et al*./2008/Sweden	24	Recombinant scFv antibody microarray	A protein signature based on 19 nonredundant analytes	A condensed set of biomarkers consisting of 19 nonredundant serum proteins differed significantly (*P* < 0.05) between cancer and normal samples
Balasenthil *et al*./2011/USA	36	ELISA	TFPI, TNC‐FN III‐C, and CA 19‐9	AUC 0.99, sensitivity 90%, and specificity 100% or sensitivity 97.2% and specificity 90%
Brand *et al*./2011/USA	160	The xMAP bead‐based technology	CA 19‐9, ICAM‐1, and OPG; CA 19‐9, CEA, and TIMP1	The panel of CA 19‐9, ICAM‐1, and OPG discriminated PDAC from HC with a sensitivity/specificity of 88/90% (AUC = 0.93) The panel of CA 19‐9, CEA, and TIMP1 discriminated PDAC from OPD subjects with a sensitivity/specificity of 76/90% (AUC = 0.85)
Nie *et al*./2014/USA	37	LC‐MS/MS analysis	19 and 25 proteins were found to show significant differences in samples between PDAC and other conditions	7 proteins considered significantly different between PDAC cases and controls, which were further validated by ELISA and lectin‐ELISA
Gerdtsson *et al*./2015/Spain	156	293‐plex recombinant antibody microarrays	PDAC *vs* others: IL‐11, cytokines, and chemokines (IL‐11, IL‐6, IL‐13, IL‐8, TNF‐α, and eotaxin), complement components (C1 inhibitor, C1q, C5, and factor B), and enzymes (HADH2, GAK, and ATP‐5B)	A multiplexed biomarker signature of up to 10 serum markers could discriminate PDAC from controls, with sensitivities and specificities in the 91–100% range (AUC = 0.98) PDAC *vs* OPD (AUC = 0.70)
Gerdtsson *et al*./2016/China	118	Recombinant antibody microarray platform	Properdin, VEGF, IL‐8, complement factor (C3), and CHP‐1	All PDAC stages could be discriminated from controls and the accuracy increased with disease progression, from stage I to stage IV (AUC): all PDAC *vs* HC 0.88; from stage I to stage IV 0.71, 0.86, 0.90, and 0.93
Sogawa *et al*./2016/Japan	80	Tandem mass tag labelling and LC‐MS/MS	C4BPA and PIGR	20 proteins were selected whose serum levels were elevated more than twofold before and after the surgery in three pairs of sera from preoperative and postoperative PDAC patients
Yoneyama *et al*./2016/Japan	164	Antibody‐based proteomics and LC‐MS/MS‐based proteomics	IGFBP2 and IGFBP3	HC *vs* IDACP: AUC for 23 proteins was greater than 0.80
Balasenthil *et al*./2017/USA	206	ELISA	TFPI, TNC‐FN III‐C, and CA 19‐9	Validation of a functional genomics‐based plasma migration signature biomarker panel
Capello *et al*./2017/USA	187	ELISA	TIMP1, LRG1, and CA 19‐9	Validation of Faca *et al*.
Liu *et al*./2017/China	80	Combined MS‐intensive methods	ApoE, ITIH3, ApoA1, and ApoL1	PDAC *vs* HC: 4 markers + CA 19‐9: sensitivity 95%, specificity 94.1%, and AUC 0.99; 4 markers: sensitivity 85%, specificity 94.1%, and AUC 0.94 PDAC *vs* HC and benign diseases: 4 markers + CA 19‐9: sensitivity 90%, specificity 75%, and AUC 0.89; 4 markers: sensitivity 85%, specificity 80%, and AUC 0.87
Park *et al*./2017/Korea	401	LC‐MS/MS	Leucine‐rich α‐2 glycoprotein, transthyretin, and CA 19‐9	Triplicate analysis to identify a 3‐panel biomarker with sensitivity over 10% greater than that of CA 19‐9 when specificity was fixed to 0.90
Park *et al*./2017/Korea	70	SIS‐MRM‐MS	Apolipoprotein A‐IV, apolipoprotein CIII, insulin‐like growth factor‐binding protein 2, and tissue inhibitor of metalloproteinase 1	The four proteins were significantly altered in PDAC cases in both the discovery and the validation phase (*P* < 0.01)
Cohen *et al*./2017/USA	221	The Bioplex 200 platform	ctDNA KRAS mutations and four proteins (CA 19‐9, CEA, hepatocyte growth factor, and osteoponin)	PDAC *vs* HC: sensitivity 64% and specificity 99.5%
Cohen *et al*./2018/USA	93	The Bioplex 200 platform	The presence of a mutation in 1933 distinct genomic positions or elevated levels of any of eight proteins (CA‐125, CEA, CA 19‐9, prolactin, hepatocyte growth factor, osteoponin, myeloperoxidase, and TIMP‐1)	PDAC *vs* HC: sensitivity 70% and specificity > 99%

References are shown in the Supporting Information.

AIP, autoimmune pancreatitis; ApoA1, apolipoprotein A‐I; ApoE, apolipoprotein E; ApoL1, apolipoprotein L1; AUC, area under the receiver operating characteristic curve; C4BPA, C4b‐binding protein α‐chain; CA 19‐9, carbohydrate antigen 19‐9; CEA, carcinoembryonic antigen; CHP‐1, calcineurin homologous protein‐1; CP, chronic pancreatitis; ctDNA, circulating tumor DNA; ELISA, enzyme‐linked immunosorbent assay; HC, healthy control; ICAM‐1, intercellular adhesion molecule 1; IDACP, invasive ductal adenocarcinoma of pancreas; IGFBP, insulin‐like growth factor‐binding protein; IL, interleukin; ITIH3, inter‐α‐trypsin inhibitor heavy chain H3; LC‐MS/MS, liquid chromatography–tandem mass spectrometry; OPD, other pancreatic disease; OPG, osteoprotegerin; PDAC, pancreatic ductal adenocarcinoma; PIGR, polymeric immunoglobulin receptor; SIS‐MRM‐MS, stable isotope dilution–multiple reaction monitoring–mass spectrometry; TFPI, tissue factor pathway inhibitor; TIMP1, tissue inhibitor of metalloproteinase 1; TNC, tenascin C; TNF‐α, tumor necrosis factor; VEGF, vascular endothelial growth factor.

**Table 3:** Study information of prospective studies of proteomics and metabolomics with PDAC

Reference	Study population	No. of cases	Platform	Identified biomarker	Diagnostic performance	Validation
Proteomics						
Nolen *et al*./2014/USA^51^	Incident PDAC cases (*n* = 135) and HC (*n* = 540) in the PLCO Screening Trial	135	Multiplexed bead‐based immunoassays	CA 19‐9, CEA, and Cyfra 21‐1	In the entire PLCO set, at 95% specificity, a panel of CA 19‐9, CEA, and Cyfra 21‐1 provided significantly elevated sensitivity of 32.4% and 29.7% in samples collected < 1 and ≥ 1 year prior to diagnosis (AUC 0.69 *vs* 0.66), respectively, compared with specificity of 25.7% and 17.2% for CA 19‐9 alone (AUC 0.68 *vs* 0.63)	A training/validation study using alternate halves of the PLCO set failed to identify a biomarker panel with significantly improved performance over CA 19‐9 alone
Mirus *et al*./2015/USA^52^	Diagnostic samples: PDAC (*n* = 24) and matched controls (*n* = 24) Pre‐diagnostic plasma: women in the WHI who would later succumb to PDAC (*n* = 87) and matched, cancer‐free controls (*n* = 87)	87	Customized antibody microarray platform	ERBB2, TNC, and ESR1	Diagnostic samples: 3‐marker panel: AUC 0.86 (0.76–0.96) 3‐marker + CA 19‐9: 0.97 (0.92–1.0)	Pre‐diagnostic cohort: AUC 0.68 (0.58–0.77), with 30% sensitivity at 90% specificity
Jenkinson *et al*./2016/UK^53^	PDAC up to 4 years prior to diagnosis (*n* = 87) and HC (*n* = 87) Validation: patients diagnosed with PDAC, chronic pancreatitis, benign biliary disease, type 2 diabetes mellitus, and HC (*n* = 298)	174	iTRAQ	TSP‐1	TSP‐1: a significant reduction in levels of TSP‐1 up to 24 months prior to diagnosis	Comparing PDAC and controls, a combination of TSP‐1 and CA 19‐9 gave an AUC of 0.85, significantly outperforming both markers alone (0.69 and 0.77) TSP‐1 also decreased in PDAC patients compared with patients with benign biliary obstruction (*P* < 0.01) Low levels of TSP‐1 correlated with poorer survival, preclinically (*P* < 0.05), and at clinical diagnosis (*P* < 0.02)
Metabolomics						
Mayers *et al*./2014/USA^54^	4 prospective cohorts (HPFS, PHS, WHI‐OS, and NHS): 453 PDAC patients and 898 matched controls	453	LC‐MS	Isoleucine, leucine, and valine	HR per SD: isoleucine 1.30 (1.15, 1.48); leucine 1.31 (1.14, 1.50); valine 1.23 (1.09, 1.39); and total 1.30 (1.14, 1.48)	NA
Nakagawa *et al*./2018/Japan^55^	A nested case–control study in the prospective JPHC: incident PDAC (*n* = 170) and matched controls (*n* = 340)	170	GC‐MS/MS	1,5‐AG and methionine	OR (Q4 *vs* Q1): 1,5‐AG 0.50 (0.27, 0.93); methionine 1.79 (0.94, 3.40)	NA
Shu *et al*./2018/China^56^	A nested case–control study in the prospective SMHS and SWHS: incident PDAC (*n* = 226) and matched controls (*n* = 226)	226	GC‐MS/UPLC‐MS	10 metabolites: Tetracosanoic acid PC (18:1/18:4) Coumarin PC (p‐18:0/22:6) PE (22:6/16:0) PS (18:0/18:0) Picolinic acid PC (15:0/18:2) PC (22:5/14:0) PE (22:6/p‐18:1)	OR per 1‐SD: 0.48 (0.36, 0.64) 0.50 (0.38, 0.66) 1.96 (1.47, 2.61) 0.53 (0.40, 0.71) 0.49 (0.35, 0.69) 0.44 (0.30, 0.66) 2.53 (1.61, 3.95) 2.32 (1.49, 3.60) 0.65 (0.51, 0.82) 0.58 (0.42, 0.79)	NA

1,5‐AG, 1,5‐anhydroglucitol; AUC, area under the receiver operating characteristic curve; CA 19‐9, carbohydrate antigen 19‐9; CEA, carcinoembryonic antigen; CP, chronic pancreatitis; ELISA, enzyme‐linked immunosorbent assay; ERBB2, v‐erb‐b2 erythroblastic leukemia viral oncogene homolog 2; ESR1, estrogen receptor 1; GC‐MS/MS, gas chromatography–tandem mass spectrometry; HC, healthy control; HPFS, Health Professionals Follow‐up Study; HR, hazard ratio; iTRAQ, isobaric tag for relative and absolute quantitation; JPHC, Japan Public Health Center‐based Prospective Study; LC‐MS, liquid chromatography–mass spectrometry; NA, not applicable; NHS, Nurses' Health Study; OR, odds ratio; PC, phosphatidylcholine; PDAC, pancreatic ductal adenocarcinoma; PE, phosphatidylethanolamine; PHS, Physicians' Health Study; PLCO, Prostate, Lung, Colorectal, and Ovarian Cancer; PS, phosphatidylserine; TNC, tenascin C; TSP‐1, thrombospondin‐1; UPLC‐MS, ultra‐performance liquid chromatography–mass spectrometry; WHI, Women's Health Initiative, WHI‐OS, Women's Health Initiative Observational Study.

At least 15 case–control studies have assessed protein panels mostly consisting of two to five biomarkers (Tables 2,S3). These case–control studies reported an area under the receiver operating characteristic curve (AUC) of 0.88–0.99 in distinguishing PDAC cases from healthy controls and of 0.82–0.90 in distinguishing PDAC cases from benign pancreatic conditions (e.g. acute pancreatitis or CP and benign pancreatic cyst).

Previous studies demonstrated the benefit of a larger panel of protein biomarkers in both Caucasians and Asians, using a case–control study design (Table S3).^49^, ^57^, ^58^, ^59^ Wingren *et al*. presented the first multiplex serum biomarker signature in a case–control study of 34 PDAC patients and 30 healthy controls, as well as 16 CP and 23 autoimmune pancreatitis patients.^49^ Based on a 25‐serum biomarker signature, an AUC of 0.95 was achieved in distinguishing PDAC from healthy controls. Of note, PDAC could be discriminated from inflammatory diseases of the pancreas (AUC: CP 0.86 and autoimmune pancreatitis 0.99). In the validation study, PDAC could also be distinguished from healthy controls and inflammatory diseases of the pancreas, achieving an AUC of 0.88. In a subsequent study, the same study group extended the platform with novel antibodies predominantly targeting cancer‐associated antigens and demonstrated robust serum signatures that could be identified in a multicenter trial.^58^ This multicenter trial involved 338 cases and control serum samples (156 PDAC, 152 other pancreatic diseases, and 30 controls with nonpancreatic conditions) from five hospitals in Spain. Based on 293‐plex recombinant antibody microarrays, PDAC cases could be distinguished from healthy participants with an AUC of 0.98, using a multiplexed biomarker signature of up to 10 serum markers.

In a recent study, the same study group identified stage‐ associated biomarkers by comparing stage I–IV patients and demonstrated the possibility for diagnosis of PDAC in earlier disease stages (Table S3).^59^ The investigators used a recombinant antibody microarray platform (350 antibodies) to analyze 213 Chinese plasma samples from PDAC patients and healthy controls. Based on a 25‐biomarker signature, they reported that all PDAC stages could be distinguished from controls with the accuracy increasing with disease progression (from stage I to stage IV). In particular, patients with stage I/II PDAC could be discriminated from healthy controls with an AUC of 0.80. Furthermore, the investigators showed a clear overlap between this study and a previous study involving Caucasians when comparing the 25 highest‐ranked antibodies, indicating that this proteomics assay was generalizable between race and ethnicity (Caucasian and Asian).

Although case–control studies have demonstrated good diagnostic accuracy of multiplex protein signatures, several prospective studies using pre‐diagnostic samples have yielded low discrimination (Table 3).^51^, ^52^, ^53^ In a case–control study with 160 PDAC cases, PDAC patients could be distinguished from healthy controls with an AUC of 0.93, using three serum protein biomarkers (CA 19‐9, intercellular adhesion molecule 1, and osteoprotegerin).^60^ However, in a population‐based prospective study of 135 incident PDAC cases, the same three‐biomarker panel merely achieved an AUC of 0.69 and 0.66 in samples collected < 1 and ≥ 1 year prior to diagnosis, not superior to the AUC for CA 19‐9 alone (0.68 *vs* 0.63).^51^ The findings suggested that this protein signature could not be used for pre‐diagnostic risk assessment. Another study analyzed plasma samples from both mouse model and diagnostic and pre‐diagnostic plasma from 87 PDAC cases in the Women's Health Initiative, an antibody microarray platform containing 130 antibodies.^52^ This cross‐species approach identified a panel of three protein biomarkers (v‐erb‐b2 erythroblastic leukemia viral oncogene homolog 2, tenascin C, and estrogen receptor 1) achieving an AUC of 0.86 in diagnostic samples. However, the AUC decreased to 0.68 in pre‐diagnostic samples (87 women who were later diagnosed with PDAC within the next 4 years of blood collection), albeit slightly superior to CA 19‐9 alone (AUC = 0.60). By contrast, a nested case–control study within the UK Collaborative Trial of Ovarian Cancer Screening with 154 PDAC cases assessed the combinations of CA 19‐9 with CA‐125 and with TSP‐1 in the pre‐diagnosis plasma samples of PDAC patients and showed good diagnostic accuracy distinguishing PDAC patients and healthy controls up to 2 years prior to diagnosis (Table 3).^47^, ^53^ The combination of TSP‐1 and CA 19‐9 achieved an AUC of 0.85, superior to both markers alone (0.69 and 0.77, respectively; *P* < 0.01).^53^

In addition to studies investigating proteomics alone, recent evidence has shown that the combination of protein biomarkers and ctDNA can reach a sensitivity of ~70% and specificity of > 99% in distinguishing PDAC cases and healthy controls (Table S3).^61^, ^62^ Combining protein biomarkers and ctDNA increases sensitivity because the majority of cancer patients are detected only by one biomarker.^61^, ^62^ Although ctDNA is elevated in 85% of patients with advanced cancers, plasma ctDNA is detectable only in a small proportion of patients with early‐stage cancers,^63^, ^64^ and the sensitivity of ctDNA tests is limited for localized cancers.^9^

### Metabolomics

Metabolomics is the comprehensive characterization of small low‐molecular‐weight metabolites in biological samples,^41^ allowing investigation of associations of metabolic alterations with conventional metabolic risk factors and with specific diseases. In recent years, metabolomics technologies (mainly mass spectrometry [MS] and nuclear magnetic resonance) have allowed the identification of metabolite biomarkers,^39^, ^41^ with the promise to inform early detection of PDAC. However, data on metabolic signatures are still limited compared with those on proteomic and genomic profiling studies of cancer. So far, there have been several case–control studies using MS metabolite profiling to examine diagnostic performance of various platforms, which measured metabolites of a diverse range (number of cases 5–360, median 49; Table S4). In general, the diagnostic performance is superior to CA 19‐9 alone and has been validated in independent test sets. However, the majority of previous studies used a case–control design and measured blood biomarkers after diagnosis of PDAC. Metabolic profiling using blood samples collected before cancer occurrence may inform early detection and improve understanding of etiology of PDAC (number of PDAC cases 170–453; Table 3).

At least 17 case–control studies suggested that MS‐based metabolomics in blood samples could be useful in PDAC detection and distinguish between PDAC from healthy controls, with an AUC greater than 0.8 (Table S4). However, only four studies reported the AUC of biomarkers to distinguish PDAC from other benign diseases (e.g. CP and biliary diseases), which were important differential diagnoses of PDAC in clinical settings. These studies showed good discrimination of PDAC cases from benign hepatobiliary disease (i.e. benign tumor and CP), CP, and type 2 diabetes. A recent study by Mayerle *et al*. used both untargeted and targeted MS‐based approaches including lipidomics and showed that PDAC patients could be distinguished from CP patients, as well as from healthy controls.^65^ Using a case–control design, 914 subjects with PDAC (*n* = 271), CP (*n* = 282), liver cirrhosis (*n* = 100), and healthy and non‐pancreatic disease controls (*n* = 261) were recruited in three consecutive studies. Of the 474 metabolites measured, a biomarker signature (nine metabolites and additionally CA 19‐9; Table S4) was identified for differentiating between PDAC and CP. The biomarker signature was successfully validated (AUC 0.94 [0.91–0.97], sensitivity 89.9% [81.0–95.5%], and specificity 91.3% [82.8–96.4%]) in a separate validation study. Of these 17 case–control studies, three studies reported on phospholipids. These studies showed that individuals with PDAC had higher sphingomyelin than individuals with CP and higher lysophosphatidylcholine than healthy controls (Table S5).

On the other hand, three nested case–control studies within prospective cohort studies investigated the performance of metabolomics in pre‐diagnostic blood samples (Table 3). Using liquid chromatography–tandem mass spectrometry, Mayers and colleagues assessed 83 metabolites in central metabolism and amino acid metabolism in 453 PDAC cases and 898 controls nested in four prospective studies.^54^ After a median follow‐up of 8 years, they found that elevated plasma levels of branched‐chain amino acids were associated with a twofold increased risk of PDAC (HR 2.00–2.13, comparing top *vs* bottom quintile). This elevated risk was independent of known predisposing factors, with the strongest association observed among subjects with samples collected 2 to 5 years before diagnosis. Another nested case–control study within the Japan Public Health Center‐based Prospective Study with 170 cases quantified 12 targeted metabolites and showed that, among patients diagnosed in the first 6 years of follow‐up, higher levels of 1,5‐anhydroglucitol (1,5‐AG), asparagine, tyrosine, and uric acid were associated with decreased risk of PDAC after adjustment for potential confounders (*P* for trend 0.02–0.04).^55^ However, when analyzing the cases during the entire follow‐up, higher 1,5‐AG and lower methionine levels showed nonsignificant associations with decreased risk of PDAC (*P* for trend 0.06 and 0.07, respectively). Recently, a nested case–control study in the Shanghai Men's Health Study and the Shanghai Women's Health Study with 226 cases identified 10 metabolites that were associated with risk of PDAC, including seven glycerophospholipids.^56^ The study also showed that the association was similar for cases diagnosed < 5 and ≥ 5 years after plasma collection. Despite the null associations of conventional lipids (triglycerides, total cholesterol, and low‐density and high‐density lipoprotein cholesterol) with PDAC in prospective studies (Table S6), these other lipids may be promising candidates as biomarkers.

## Risk prediction

Lifestyle and metabolic risk factors, as well as genomics and blood‐based biomarkers, are predictive of risk of PDAC. However, blood‐based biomarkers have been rarely investigated in relation to risk prediction in the general population as the majority of previous studies are hospital‐based case–control studies. The primary goal of risk prediction model is to develop a tool for identifying individuals at a high risk of PDAC. Well‐developed models can provide accurate risk assessment for individuals and can inform screening decisions. Although imaging (endoscopic ultrasonography and/or magnetic resonance cholangiopancreatography) has been recommended for initial screening among high‐risk populations, there is no consensus on screening modalities and intervals for follow‐up imaging.^1^, ^2^, ^3^ There are two types of risk prediction models, developed either in high‐risk populations (e.g. positive family history of PDAC and newly onset diabetes) or in the general population (Table 4).

**Table 4:** Study information of PDAC risk prediction

Reference	Study population	No. of cases	Predictor	Diagnostic performance in the training set	Diagnostic performance in the validation set
High‐risk population					
Risch *et al*./2015/USA^67^	A representative case–control study in Connecticut including 362 newly diagnosed PDAC cases and 690 matched controls	362	Current smoking, current use of PPI, anti‐heartburn medications, recent diagnosis of diabetes, recent diagnosis of pancreatitis, Jewish ancestry, and non‐O blood group	5‐year absolute risk calculated from the SEER data†: 0.87% of controls that had combinations of all these factors had 5‐year absolute risks > 5%	NA
Boursi *et al*./2017/UK^68^	109 385 individuals with incident diabetes after the age of 35 years and 3 or more years of follow‐up after diagnosis of diabetes	390	Age, BMI, change in BMI, smoking, proton pump inhibitors, antidiabetic medications, hemoglobin A1C, cholesterol, hemoglobin, creatinine, and alkaline phosphatase	AUC: 0.82 (95% CI 0.75, 0.89) Hosmer–Lemeshow goodness‐of‐fit test: *P* values 0.10–0.78 If the predicted risk threshold for definitive PDAC screening was set at 1% over 3 years: sensitivity 44.7%, specificity 94.0%, and positive predictive value 2.6%	Internal validation by bootstrap: negligible optimism according to Harrell's algorithm: 0.0003 (95% CI −0.0057, 0.0057)
General population					
Kim *et al*./2004/USA^69^	738 953 men over a period of 10‐year follow‐up in the Health Professionals' Follow‐up Study	96	Smoking, history of diabetes, and vegetables consumption	Validation of the Harvard Cancer Risk Index	Discrimination: AUC 0.72 (0.67, 0.77) Calibration: O/E ratios across Risk Index RR categories 2.10 to ≤ 5.10: 4.91 (0, 10.46) 1.10 to ≤ 2.10: 2.16 (0.94, 3.39) 0.90 to ≤ 1.10: 2.03 (0.40, 3.65) 0.50 to ≤ 0.90: 0.90 (0.70, 1.11)
Klein *et al*./2013/USA, Europe^70^	3349 cases and 3654 controls from the PanScan I–III Consortium of European ancestry (12 nested case–control studies and 8 case–control studies)	3349	Smoking, heavy alcohol use, obesity, diabetes > 3 years, family history of PDAC, and non‐O ABO genotype	AUC: nongenetic factors 0.58 (0.56, 0.60), genetic factors 0.57 (0.55, 0.59), and both nongenetic and genetic factors 0.61 (0.58, 0.63) Fewer than 0.3% US non‐Hispanic whites had more than a 5% predicted lifetime absolute risk	NA
Hippisley‐Cox and Coupland/2015/UK^71^	Routinely collected data from 753 QResearch general practices in England: 4.96 million patients aged 25–84 years in the derivation cohort and 1.64 million in the validation cohort	7119	Men: age, BMI, Townsend score, smoking, chronic pancreatitis, diabetes, and blood cancer Women: age, BMI, Townsend score, smoking, chronic pancreatitis, diabetes, renal cancer, and breast cancer	NA	Discrimination: AUC men 0.857 (95% CI 0.847, 0.867); AUC women 0.865 (0.855, 0.875) Calibration: Close correspondence was observed between the mean predicted risks and the observed risks within each model tenth Women: 10‐year predicted risk top 10% (> 0.50), sensitivity 50%, specificity 90%, and observed risk at 10 years 0.76%; men: (> 0.52) sensitivity 49%, specificity 90%, and observed risk at 10 years 0.86%
Yu *et al*./2016/Korea^72^	1 289 933 men and 557 701 women in Korea who had biennial examinations in 1996–1997 with an 8‐year follow‐up and 500 046 men and 627 629 women who had biennial examinations in 1998–1999 in the validation cohort	2195	Men: age, height, BMI, fasting glucose, urine glucose, smoking, and age at smoking initiation Women: height, BMI, fasting glucose, urine glucose, smoking, and drinking	NA	Discrimination: AUC: men 0.813 (95% CI 0.800, 0.826); AUC women 0.804 (0.788, 0.820) Calibration: Hosmer–Lemeshow *χ* ^2^ statistics: men 7.478 (*P* = 0.587); women 10.297 (*P* = 0.327) Among participants of the top quintile of the estimated probability of developing PDAC, the cumulative probabilities at 10 years were 0.359% (0.335, 0.384) in men and 0.292% (0.261, 0.327) in women

The O/E ratio represents the age‐standardized incidence ratio for the group of individuals within the Risk Index RR category, standardized using observed 10‐year age‐specific incidence rates in the cohort. In all studies, diagnosis of PDAC was ascertained by medical records and the International Classification of Diseases code.

† The SEER data provide the average age‐specific and sex‐specific probabilities of developing PDAC for populations covered by the SEER registries.

AUC, area under the receiver operating characteristic curve; BMI, body mass index; CI, confidence interval; NA, not applicable; PDAC, pancreatic ductal adenocarcinoma; PPI, proton‐pump inhibitor; RR, relative risk; SEER, Surveillance Epidemiology and End Results.

PancPRO is a Mendelian model for PDAC risk prediction for identifying high‐risk individuals in those with familial PDAC, built using a Bayesian modeling framework, and was validated using an independent cohort in the National Family Pancreas Tumor Registry (961 families and 26 incident PDAC cases).^66^ PancPRO was shown to have good discrimination and calibration in the independent validation cohort, with an AUC of 0.75 (0.68–0.81) and an observed to predicted PDAC ratio of 0.83 (0.52–1.20). Another risk prediction model included detectable symptomatology preceding the diagnosis of PDAC as well as other risk factors.^67^ The estimates were obtained from a case–control study where information on current medications and recent signs and symptoms was collected. The 5‐year absolute risk was calculated from the US Surveillance Epidemiology and End Results incidence data from 2008 to 2010. A total of 0.87% of controls had 5‐year absolute risks > 5% who had a combination of recent diagnosis of diabetes and pancreatitis, current use of proton‐pump inhibitors, Jewish ancestry, non‐O blood group, and current smoking. A recent study developed and internally validated a risk prediction model for PDAC among patients with newly onset diabetes, showing the promise of risk stratification among high‐risk populations.^68^ The study involved 109, 385 individuals with newly diagnosed diabetes, and the outcome was PDAC diagnosed within 3 years of diabetes onset. The prediction model included demographic, behavioral, and clinical variables that were routinely collected at the time of diabetes diagnosis. They showed that if the predicted risk threshold was set at 1% over 3 years, the model would have a sensitivity of 44.7%, specificity of 94.0%, and a positive predictive value of 2.6%.

For risk prediction models developed in the general population, the majority of previous studies developed risk prediction models using routinely collected data and traditional regression models (Table 4),^69^, ^70^, ^71^, ^72^ and some validated the prediction models in separate populations^71^, ^72^ and showed good discrimination and calibration. Two retrospective cohort studies have developed and validated prediction models for PDAC using information on socio‐demographic, lifestyle, and clinical variables,^71^, ^72^ and both have shown good diagnostic accuracy in independent cohorts. The first study included routinely collected data of ~5 million patients aged 25–84 years from 753 QResearch general practices in England.^71^ In an external validation cohort of 1.6 million patients, their sex‐specific risk prediction model showed good diagnostic accuracy (AUC of 0.86 in men and 0.87 in women) and showed a 10‐year absolute risk of ~0.8% in both sexes for participants with the top 10% of predicted risk. Likewise, another retrospective cohort involving ~2 million Korean individuals who underwent biennial examinations reported an 8‐year absolute risk for participants with all risk factors included in the prediction model (1.5% in men and 1.2% in women).^72^ So far, only the PanScan Consortium developed an RR model involving genetic risk factors as well as traditional, nongenetic risk factors.^70^ However, their model had limited diagnostic accuracy, with an AUC of 0.58 for nongenetic factors, 0.57 for genetic factors, and 0.61 for both nongenetic and genetic factors. In particular, they found that the genetic factors did not add substantively to a risk model based on lifestyle factors only.

## Conclusions and future directions

Despite the large number of risk factors suggested by observational studies, the magnitude of RRs is overall small and the PAFs are low. Risk prediction models incorporating these lifestyle and metabolic risk factors as well as other factors routinely collected by health insurance systems have achieved satisfying sensitivity and specificity. Genetic studies of very large samples sizes are required for the discovery of genetic variants ranging from rare, low‐frequency, and common variants in order to inform risk prediction models. Novel blood‐based biomarkers, particularly proteomics and metabolomics, can inform early diagnosis of PDAC. However, prospective cohort studies that collect pre‐diagnostic samples and validation in independent studies are warranted. In this context, prospective biobank studies with samples collected prior to disease onset are valuable resources. For example, the China Kadoorie Biobank has proposed a multi‐omic approach (metabolomics, proteomics, and genomics) to investigate novel biomarkers relevant for risk prediction and early diagnosis of PDAC. Similarly, the European Prospective Investigation into Cancer and Nutrition has included metabolomics in their ongoing research topics for PDAC.

Because of the lack of noninvasive and low‐cost screening tools, the current recommendation is that screening the general population for PDAC is not feasible, and screening will need to be restricted to people at high risk of PDAC.^73^ Based on our review of literature in this field, we propose that a bridge between risk factor epidemiology and multi‐omics investigations is needed because (i) combinations of biomarkers, as well as combination of biomarkers with traditional risk factors and genomics data, can provide much more information than a single biomarker alone (e.g. CA 19‐9); (ii) developing algorithms to identify high‐risk populations and biomarkers with sufficient discriminatory power that are cost‐effective are needed to inform clinical decisions; and (iii) multi‐omics investigations can help identify etiological factors for PDAC and new pathways for potential therapeutic targets for treatment.

## Supporting information


**Table S1.** Reviews of ctDNA, cell‐free non‐coding RNA, exosomes, microbiome, circulating tumor cells, and PDAC.
**Table S2.** Study information of GWAS studies of PDAC.
**Table S3.** Study information of case–control studies of proteomics and PDAC.
**Table S4.** Study information of case–control studies of metabolomics and PDAC.
**Table S5.** Case–control studies of metabolomics and PDAC reporting on phospholipids.
**Table S6.** Prospective studies of blood lipids and risk of PDAC.

## References

[1] Hidalgo M . Pancreatic cancer. N. Engl. J. Med. 2010; 362: 1605–1617. 20427809 10.1056/NEJMra0901557

[2] Yadav D , Lowenfels AB . The epidemiology of pancreatitis and pancreatic cancer. Gastroenterology 2013; 144: 1252–1261. 23622135 10.1053/j.gastro.2013.01.068 PMC3662544

[3] Harsha H , Kandasamy K , Ranganathan P *et al* A compendium of potential biomarkers of pancreatic cancer. PLoS Med. 2009; 6: e1000046. 19360088 10.1371/journal.pmed.1000046 PMC2661257

[4] Gillen S , Schuster T , Meyer Zum Buschenfelde C , Friess H , Kleeff J . Preoperative/neoadjuvant therapy in pancreatic cancer: a systematic review and meta‐analysis of response and resection percentages. PLoS Med. 2010; 7: e1000267. 20422030 10.1371/journal.pmed.1000267 PMC2857873

[5] Cancer Research UK . Survival statistics for pancreatic cancer. 2017 http://www.myendnoteweb.com/EndNoteWeb.html (accessed July 25 2017).

[6] Siegel RL , Miller KD , Jemal A . Cancer statistics, 2016. CA Cancer J. Clin. 2016; 66: 7–30. 26742998 10.3322/caac.21332

[7] Yachida S , Jones S , Bozic I *et al* Distant metastasis occurs late during the genetic evolution of pancreatic cancer. Nature 2010; 467: 1114–1117. 20981102 10.1038/nature09515 PMC3148940

[8] Zhou B , Xu JW , Cheng YG *et al* Early detection of pancreatic cancer: where are we now and where are we going? Int. J. Cancer 2017; 141: 231–241. 28240774 10.1002/ijc.30670

[9] Zhang X , Shi S , Zhang B , Ni Q , Yu X , Xu J . Circulating biomarkers for early diagnosis of pancreatic cancer: facts and hopes. Am. J. Cancer Res. 2018; 8: 332–353. 29636993 PMC5883088

[10] Archibugi L , Signoretti M , Capurso G . The microbiome and pancreatic cancer: an evidence‐based association? J. Clin. Gastroenterol. 2018; 52 10.1097/MCG.0000000000001092. 30001289

[11] Maisonneuve P , Lowenfels AB . Risk factors for pancreatic cancer: a summary review of meta‐analytical studies. Int. J. Epidemiol. 2015; 44: 186–198. 25502106 10.1093/ije/dyu240

[12] Iodice S , Gandini S , Maisonneuve P , Lowenfels AB . Tobacco and the risk of pancreatic cancer: a review and meta‐analysis. Langenbecks Arch. Surg. 2008; 393: 535–545. 18193270 10.1007/s00423-007-0266-2

[13] Tramacere I , Scotti L , Jenab M *et al* Alcohol drinking and pancreatic cancer risk: a meta‐analysis of the dose–risk relation. Int. J. Cancer 2010; 126: 1474–1486. 19816941 10.1002/ijc.24936

[14] World Cancer Research Fund . Food, nutrition, physical activity, and the prevention of pancreatic cancer. 2012 http://www.aicr.org/continuous‐update‐project/reports/pancreatic‐cancer‐2012‐report.pdf (accessed 15 May 2018).

[15] Behrens G , Jochem C , Schmid D , Keimling M , Ricci C , Leitzmann MF . Physical activity and risk of pancreatic cancer: a systematic review and meta‐analysis. Eur. J. Epidemiol. 2015; 30: 279–298. 25773752 10.1007/s10654-015-0014-9

[16] Aune D , Greenwood D , Chan D *et al* Body mass index, abdominal fatness and pancreatic cancer risk: a systematic review and non‐linear dose–response meta‐analysis of prospective studies. Ann. Oncol. 2011; 23: 843–852. 21890910 10.1093/annonc/mdr398

[17] Pang Y , Holmes MV , Kartsonaki C *et al* Young adulthood and adulthood adiposity in relation to incidence of pancreatic cancer: a prospective study of 0.5 million Chinese adults and a meta‐analysis. J. Epidemiol. Community Health 2017; 71: 1059–1067. 28900029 10.1136/jech-2017-208895 PMC5847093

[18] Pang Y , Kartsonaki C , Guo Y *et al* Diabetes, plasma glucose and incidence of pancreatic cancer: a prospective study of 0.5 million Chinese adults and a meta‐analysis of 22 cohort studies. Int. J. Cancer 2017; 140: 1781–1788. 28063165 10.1002/ijc.30599 PMC5396360

[19] Duell E , Lucenteforte E , Olson S *et al* Pancreatitis and pancreatic cancer risk: a pooled analysis in the International Pancreatic Cancer Case‐Control Consortium (PanC4). Ann. Oncol. 2012; 23: 2964–2970. 22767586 10.1093/annonc/mds140 PMC3477881

[20] Carreras‐Torres R , Johansson M , Gaborieau V *et al* The role of obesity, type 2 diabetes, and metabolic factors in pancreatic cancer: a Mendelian randomization study. J. Natl. Cancer Inst. 2017; 109. 10.1093/jnci/djx012 PMC5721813 28954281

[21] Sah RP , Nagpal SJ , Mukhopadhyay D , Chari ST . New insights into pancreatic cancer-induced paraneoplastic diabetes. Nat Rev Gastroenterol Hepatol 2013; 10: 423–433. 23528347 10.1038/nrgastro.2013.49 PMC3932322

[22] Pannala R , Basu A , Petersen GM , Chari ST . New‐onset diabetes: a potential clue to the early diagnosis of pancreatic cancer. Lancet Oncol. 2009; 10: 88–95. 19111249 10.1016/S1470-2045(08)70337-1 PMC2795483

[23] Pannala R , Leirness JB , Bamlet WR , Basu A , Petersen GM , Chari ST . Prevalence and clinical profile of pancreatic cancer‐associated diabetes mellitus. Gastroenterology 2008; 134: 981–987. 18395079 10.1053/j.gastro.2008.01.039 PMC2323514

[24] Davey Smith G , Ebrahim S . ‘Mendelian randomization’: can genetic epidemiology contribute to understanding environmental determinants of disease? Int. J. Epidemiol. 2003; 32: 1–22. 12689998 10.1093/ije/dyg070

[25] Stolzenberg‐Solomon RZ , Limburg P , Pollak M , Taylor PR , Virtamo J , Albanes D . Insulin‐like growth factor (IGF)‐1, IGF‐binding protein‐3, and pancreatic cancer in male smokers. Cancer Epidemiol. Prev. Biomarkers 2004; 13: 438–444. 15006921

[26] Stolzenberg‐Solomon RZ , Graubard BI , Chari S *et al* Insulin, glucose, insulin resistance, and pancreatic cancer in male smokers. JAMA 2005; 294: 2872–2878. 16352795 10.1001/jama.294.22.2872

[27] Wolpin B , Michaud D , Giovannucci E *et al* Circulating insulin‐like growth factor axis and the risk of pancreatic cancer in four prospective cohorts. Br. J. Cancer 2007; 97: 98. 17533398 10.1038/sj.bjc.6603826 PMC2359655

[28] Wolpin BM , Michaud DS , Giovannucci EL *et al* Circulating insulin‐like growth factor binding protein‐1 and the risk of pancreatic cancer. Cancer Res. 2007; 67: 7923–7928. 17699799 10.1158/0008-5472.CAN-07-0373

[29] Douglas JB , Silverman DT , Pollak MN , Tao Y , Soliman AS , Stolzenberg‐Solomon RZ . Serum IGF‐I, IGF‐II, IGFBP‐3, and IGF‐I/IGFBP‐3 molar ratio and risk of pancreatic cancer in the prostate, lung, colorectal, and ovarian cancer screening trial. Cancer Epidemiol. Prev. Biomarkers 2010; 19: 2298–2306. 10.1158/1055-9965.EPI-10-0400 PMC2936681 20699371

[30] Rohrmann S , Grote V , Becker S *et al* Concentrations of IGF‐I and IGFBP‐3 and pancreatic cancer risk in the European Prospective Investigation into Cancer and Nutrition. Br. J. Cancer 2012; 106: 1004. 22315049 10.1038/bjc.2012.19 PMC3305958

[31] Wolpin BM , Bao Y , Qian ZR *et al* Hyperglycemia, insulin resistance, impaired pancreatic β‐cell function, and risk of pancreatic cancer. J. Natl. Cancer Inst. 2013; 105: 1027–1035. 23847240 10.1093/jnci/djt123 PMC3714020

[32] Maisonneuve P , Amar S , Lowenfels AB . Periodontal disease, edentulism, and pancreatic cancer: a meta‐analysis. Ann. Oncol. 2017; 28: 985–995. 28453689 10.1093/annonc/mdx019

[33] Bao Y , Giovannucci EL , Kraft P *et al* Inflammatory plasma markers and pancreatic cancer risk: a prospective study of five U.S. cohorts. Cancer Epidemiol. Biomarkers Prev. 2013; 22: 855–861. 23462920 10.1158/1055-9965.EPI-12-1458 PMC3650127

[34] Douglas JB , Silverman DT , Weinstein SJ *et al* Serum C‐reactive protein and risk of pancreatic cancer in two nested, case–control studies. Cancer Epidemiol. Biomarkers Prev. 2011; 20: 359–369. 21173171 10.1158/1055-9965.EPI-10-1024 PMC3495286

[35] Grote VA , Kaaks R , Nieters A *et al* Inflammation marker and risk of pancreatic cancer: a nested case–control study within the EPIC cohort. Br. J. Cancer 2012; 106: 1866–1874. 22617158 10.1038/bjc.2012.172 PMC3364108

[36] Low SK , Kuchiba A , Zembutsu H *et al* Genome‐wide association study of pancreatic cancer in Japanese population. PLoS One 2010; 5: e11824. 20686608 10.1371/journal.pone.0011824 PMC2912284

[37] Wu C , Miao X , Huang L *et al* Genome‐wide association study identifies five loci associated with susceptibility to pancreatic cancer in Chinese populations. Nat. Genet. 2011; 44: 62–66. 22158540 10.1038/ng.1020

[38] Chatterjee N , Shi J , García‐Closas M . Developing and evaluating polygenic risk prediction models for stratified disease prevention. Nat. Rev. Genet. 2016; 17: 392–406. 27140283 10.1038/nrg.2016.27 PMC6021129

[39] Soininen P , Kangas AJ , Wurtz P , Suna T , Ala‐Korpela M . Quantitative serum nuclear magnetic resonance metabolomics in cardiovascular epidemiology and genetics. Circ. Cardiovasc. Genet. 2015; 8: 192–206. 25691689 10.1161/CIRCGENETICS.114.000216

[40] Solier C , Langen H . Antibody‐based proteomics and biomarker research—current status and limitations. Proteomics 2014; 14: 774–783. 24520068 10.1002/pmic.201300334

[41] Tzoulaki I , Ebbels TM , Valdes A , Elliott P , Ioannidis JP . Design and analysis of metabolomics studies in epidemiologic research: a primer on‐omic technologies. Am. J. Epidemiol. 2014; 180: 129–139. 24966222 10.1093/aje/kwu143

[42] Borrebaeck CA . Precision diagnostics: moving towards protein biomarker signatures of clinical utility in cancer. Nat. Rev. Cancer 2017; 17: 199–204. 28154374 10.1038/nrc.2016.153

[43] Locker GY , Hamilton S , Harris J *et al* ASCO 2006 update of recommendations for the use of tumour markers in gastrointestinal cancer. J. Clin. Oncol. 2006; 24: 5313–5327. 17060676 10.1200/JCO.2006.08.2644

[44] Poruk KE , Gay DZ , Brown K *et al* The clinical utility of CA 19‐9 in pancreatic adenocarcinoma: diagnostic and prognostic updates. Curr. Mol. Med. 2013; 13: 340–351. 23331006 10.2174/1566524011313030003 PMC4419808

[45] Goonetilleke K , Siriwardena A . Systematic review of carbohydrate antigen (CA 19‐9) as a biochemical marker in the diagnosis of pancreatic cancer. Eur. J. Surg. Oncol. 2007; 33: 266–270. 17097848 10.1016/j.ejso.2006.10.004

[46] Duffy M , Sturgeon C , Lamerz R *et al* Tumour markers in pancreatic cancer: a European Group on Tumour Markers (EGTM) status report. Ann. Oncol. 2009; 21: 441–447. 19690057 10.1093/annonc/mdp332

[47] O'Brien DP , Sandanayake NS , Jenkinson C *et al* Serum CA19‐9 is significantly upregulated up to 2 years before diagnosis with pancreatic cancer: implications for early disease detection. Clin. Cancer Res. 2015; 21: 622–631. 24938522 10.1158/1078-0432.CCR-14-0365 PMC4181906

[48] Bunger S , Laubert T , Roblick UJ , Habermann JK . Serum biomarkers for improved diagnostic of pancreatic cancer: a current overview. J. Cancer Res. Clin. Oncol. 2011; 137: 375–389. 21193998 10.1007/s00432-010-0965-x PMC11827947

[49] Wingren C , Sandstrom A , Segersvard R *et al* Identification of serum biomarker signatures associated with pancreatic cancer. Cancer Res. 2012; 72: 2481–2490. 22589272 10.1158/0008-5472.CAN-11-2883

[50] Kaur S , Smith LM , Patel A *et al* A combination of MUC5AC and CA19‐9 improves the diagnosis of pancreatic cancer: a multicenter study. Am. J. Gastroenterol. 2017; 112: 172–183. 27845339 10.1038/ajg.2016.482 PMC5365072

[51] Nolen BM , Brand RE , Prosser D *et al* Prediagnostic serum biomarkers as early detection tools for pancreatic cancer in a large prospective cohort study. PLoS One 2014; 9: e94928. 24747429 10.1371/journal.pone.0094928 PMC3991628

[52] Mirus JE , Zhang Y , Li CI *et al* Cross‐species antibody microarray interrogation identifies a 3‐protein panel of plasma biomarkers for early diagnosis of pancreas cancer. Clin. Cancer Res. 2015; 21: 1764–1771. 25589628 10.1158/1078-0432.CCR-13-3474 PMC4391639

[53] Jenkinson C , Elliott VL , Evans A *et al* Decreased serum thrombospondin‐1 levels in pancreatic cancer patients up to 24 months prior to clinical diagnosis: association with diabetes mellitus. Clin. Cancer Res. 2016; 22: 1734–1743. 26573598 10.1158/1078-0432.CCR-15-0879 PMC4820087

[54] Mayers JR , Wu C , Clish CB *et al* Elevation of circulating branched‐chain amino acids is an early event in human pancreatic adenocarcinoma development. Nat. Med. 2014; 20: 1193–1198. 25261994 10.1038/nm.3686 PMC4191991

[55] Nakagawa T , Kobayashi T , Nishiumi S *et al* Metabolome analysis for pancreatic cancer risk in nested case–control study: JPHC Study. Cancer Sci. 2018; 109: 1672. 29575390 10.1111/cas.13573 PMC5980145

[56] Shu X , Zheng W , Yu D *et al* Prospective metabolomics study identifies potential novel blood metabolites associated with pancreatic cancer risk. Int. J. Cancer 2018 May 1 10.1002/ijc.31574. PMC6195470 29717485

[57] Ingvarsson J , Wingren C , Carlsson A *et al* Detection of pancreatic cancer using antibody microarray‐based serum protein profiling. Proteomics 2008; 8: 2211–2219. 18528842 10.1002/pmic.200701167

[58] Gerdtsson AS , Malats N , Sall A *et al* A multicenter trial defining a serum protein signature associated with pancreatic ductal adenocarcinoma. Int. J. Proteomics 2015; 2015: 587250. 26587286 10.1155/2015/587250 PMC4637476

[59] Gerdtsson AS , Wingren C , Persson H *et al* Plasma protein profiling in a stage defined pancreatic cancer cohort—implications for early diagnosis. Mol. Oncol. 2016; 10: 1305–1316. 27522951 10.1016/j.molonc.2016.07.001 PMC5423191

[60] Brand RE , Nolen BM , Zeh HJ *et al* Serum biomarker panels for the detection of pancreatic cancer. Clin. Cancer Res. 2011; 17: 805–816. 21325298 10.1158/1078-0432.CCR-10-0248 PMC3075824

[61] Cohen JD , Javed AA , Thoburn C *et al* Combined circulating tumour DNA and protein biomarker‐based liquid biopsy for the earlier detection of pancreatic cancers. Proc. Natl. Acad. Sci. U. S. A. 2017; 114: 10202–10207. 28874546 10.1073/pnas.1704961114 PMC5617273

[62] Cohen JD , Li L , Wang Y *et al* Detection and localization of surgically resectable cancers with a multi‐analyte blood test. Science 2018; 359: 926–930. 29348365 10.1126/science.aar3247 PMC6080308

[63] Bettegowda C , Sausen M , Leary RJ *et al* Detection of circulating tumour DNA in early‐ and late‐stage human malignancies. Sci. Transl. Med. 2014; 6: 224ra24. 10.1126/scitranslmed.3007094 PMC4017867 24553385

[64] Wang Y , Springer S , Mulvey CL *et al* Detection of somatic mutations and HPV in the saliva and plasma of patients with head and neck squamous cell carcinomas. Sci. Transl. Med. 2015; 7: 293ra104. 10.1126/scitranslmed.aaa8507 PMC4587492 26109104

[65] Mayerle J , Kalthoff H , Reszka R *et al* Metabolic biomarker signature to differentiate pancreatic ductal adenocarcinoma from chronic pancreatitis. Gut 2018; 67: 128–137. 28108468 10.1136/gutjnl-2016-312432 PMC5754849

[66] Wang W , Chen S , Brune KA , Hruban RH , Parmigiani G , Klein AP . PancPRO: risk assessment for individuals with a family history of pancreatic cancer. J. Clin. Oncol. 2007; 25: 1417–1422. 17416862 10.1200/JCO.2006.09.2452 PMC2267288

[67] Risch HA , Yu H , Lu L , Kidd MS . Detectable symptomatology preceding the diagnosis of pancreatic cancer and absolute risk of pancreatic cancer diagnosis. Am. J. Epidemiol. 2015; 182: 26–34. 26049860 10.1093/aje/kwv026 PMC4479115

[68] Boursi B , Finkelman B , Giantonio BJ *et al* A clinical prediction model to assess risk for pancreatic cancer among patients with new‐onset diabetes. Gastroenterology 2017; 152: 840–850 e3. 27923728 10.1053/j.gastro.2016.11.046 PMC5337138

[69] Kim DJ , Rockhill B , Colditz GA . Validation of the Harvard Cancer Risk Index: a prediction tool for individual cancer risk. J. Clin. Epidemiol. 2004; 57: 332–340. 15135833 10.1016/j.jclinepi.2003.08.013

[70] Klein AP , Lindstrom S , Mendelsohn JB *et al* An absolute risk model to identify individuals at elevated risk for pancreatic cancer in the general population. PLoS One 2013; 8: e72311. 24058443 10.1371/journal.pone.0072311 PMC3772857

[71] Hippisley‐Cox J , Coupland C . Development and validation of risk prediction algorithms to estimate future risk of common cancers in men and women: prospective cohort study. BMJ Open 2015; 5: e007825. 10.1136/bmjopen-2015-007825 PMC4368998 25783428

[72] Yu A , Woo SM , Joo J *et al* Development and validation of a prediction model to estimate individual risk of pancreatic cancer. PLoS One 2016; 11: e0146473. 26752291 10.1371/journal.pone.0146473 PMC4708985

[73] Canto MI , Harinck F , Hruban RH *et al* International Cancer of the Pancreas Screening (CAPS) Consortium summit on the management of patients with increased risk for familial pancreatic cancer. Gut 2013; 62: 339–347. 23135763 10.1136/gutjnl-2012-303108 PMC3585492

