# Supplementary material for: A review of lifestyle, metabolic risk factors, and blood‐based biomarkers for early diagnosis of pancreatic ductal adenocarcinoma
Source: J Gastroenterol Hepatol. 2019 Jan 17;34(2):330–45. doi: 10.1111/jgh.14576 (PMC6378598; doi:10.1111/jgh.14576)
Supplement: Supplementary file 1 — Table S1. Reviews of ctDNA, cell‐free non‐coding RNA, exosomes, microbiome, circulating tumor cells, and PDAC. Table S2. Study information of GWAS studies of PDAC. Table S3. Study information of case–control studies of proteomics and PDAC. Table S4. Study information of case–control studies of metabolomics and PDAC. Table S5. Case–control studies of metabolomics and PDAC reporting on phospholipids. Table S6. Prospective studies of blood lipids and risk of PDAC. [file JGH-34-330-s001.docx]

**Supplementary Material**

Table of Contents

[Supplementary Table 1. Reviews of ctDNA, cell-free non-coding RNA, exosomes, microbiome, circulating tumour cells, and PDAC 3](#_Toc531781774)

[Supplementary Table 2. Study information of GWAS studies of PDAC 4](#_Toc531781775)

[Supplementary Table 3. Study information of case-control studies of proteomics and PDAC 6](#_Toc531781776)

[Supplementary Table 4. Study information of case-control studies of metabolomics and PDAC 10](#_Toc531781777)

[Supplementary Table 5. Case-control studies of metabolomics and PDAC reporting on phospholipids 13](#_Toc531781778)

[Supplementary Table 6. Prospective studies of blood lipids and risk of PDAC 14](#_Toc531781779)

# Supplementary Table 1. Reviews of ctDNA, cell-free non-coding RNA, exosomes, microbiome, circulating tumour cells, and PDAC

|  | **Biomarkers** |
| --- | --- |
| 1. ctDNA^1^ | KRAS |
|  |  |
| 2. Epigenetic modification of ctDNA^2^ | UCHL1, NPTX2, SARP2, CLDN5, FOXE1, CDH3 |
|  |  |
| 3. Cell-free noncoding RNA^1,2^ | miRNA: miR-486-5p, miR-938, miR-126-3p, miR-106b-3p, miR-223, miR-182, miR-10b, miR-30C, miR-106b, miR-155, miR-212, miR-145, miR-150, miR-223, miR-636  lncRNA: H19, HOTAIR, HOTTIP, MALAT-1, HULC, GAS5, HDRF, RDRF |
|  |  |
| 4. Exosomes^2^ | Glypican-1, migration inhibitory factor, αvβ5, α6β4, α6β1 |
|  |  |
| 5. Microbiome^3^ | A muciniphila, Actinobacteria, Bacteroides, Bacillaceae, butyrate producing bacteria, Clostridiaceae, Clostridium, Enterobacteriaceae, Firmicutes, Proteobacteria |
| 6. Circulating tumour cells^4^ | Detection rate:  All stages: 18-78.6%  Early stage: 57.5%  Advanced stage: 11-94.4% |
|  | Pre-operative: 33.8-75%  Intra-operative: 61.9-73.3% |

# Supplementary Table 2. Study information of GWAS studies of PDAC

| **Reference** | **Study population** | **No. of cases** | **SNP** | **Chromosome** | **Gene** | **Novelty** | **OR** | ***P*-value** |
| --- | --- | --- | --- | --- | --- | --- | --- | --- |
| Low/2010^5^ | 991 invasive PDAC cases and 5209 controls of Japanese ancestry | 991 | rs9502893 | 6p25.3 | FOXQ1 | New | 1.29 (1.17, 1.43) | 3.30x10^-7^ |
|  |  |  | rs708224 | 12p11.21 | BICD1 | New | 1.32 (1.19, 1.47) | 3.30x10^-7^ |
|  |  |  | rs6464375 | 7q36.2 | DPP6 transcript variant 3 | New | 3.73 (2.24, 6.21) | 4.41x10^-7^ |
| Petersen/2010^6^ | PanScan I and PanC4: 3851 PDAC cases and 3934 controls of European, Chinese, and other ancestry | 3851 | rs9543325 | 13q22.1 | Non-genic region | New | 1.26 (1.18, 1.35) | 3.27x10^-11^ |
|  |  |  | rs9564966 |  |  | New | 1.21 (1.13, 1.30) | 5.86x10^-8^ |
|  |  |  | rs3790844 | 1q32.1 | NR5A2 | New | 0.77 (0.71, 0.84) | 2.45x10^-10^ |
|  |  |  | rs10919791 |  |  | New | 0.77 (0.71, 0.84) | 6.37x10^-10^ |
|  |  |  | rs3790843 |  |  | New | 0.81 (0.75, 0.87) | 6.69x10^-9^ |
|  |  |  | rs12029406 |  |  | New | 0.83 (0.78, 0.89) | 1.04x10^-7^ |
|  |  |  | rs4465241 |  |  | New | 1.25 (1.14, 1.37) | 4.79x10^-7^ |
|  |  |  | rs401681 | 5p15.33 | CLPTM1L | New | 1.19 (1.11, 1.27) | 3.66x10^-7^ |
| Wu/2012^7^ | 3584 PDAC cases and 4868 controls of Chinese ancestry | 3584 | rs372883 | 21q21.3 | BACH1 | New | 0.79 (0.75, 0.84) | 2.24X10^-13^ |
|  |  |  | rs2255280 | 5p13.1 | DAB2 | New | 0.81 (0.76, 0.87) | 4.18X10^-10^ |
|  |  |  | rs1547374 | 21q22.3 | TGG1 | New | 0.79 (0.75, 0.84) | 3.71x10^-13^ |
|  |  |  | rs5768709 | 22q13.32 | FAM19A5 | New | 1.25 (1.17, 1.34) | 1.41x10^-10^ |
|  |  |  | rs12413624 | 10q26.11 | PRLHR | New | 1.23 (1.16, 1.31) | 5.12x10^-11^ |
|  |  |  | rs4885093 | 13q22.1 | NA | Replicated | 1.25 (1.18, 1.33) | 1.57x10^-12^ |
| Wolpin/2014^8^ | PanScan III: 7683 PDAC cases and 14,397 controls of European ancestry | 7683 | rs6971499 | 7q32.2 | LINC-PINT | New | 0.79 (0.74, 0.84) | 3.0x10^-12^ |
|  |  |  | rs7190458 | 16q23.1 | BCAR1/CTRB1/CTRB2 | New | 1.46 (1.30, 1.65) | 1.1x10^-10^ |
|  |  |  | rs9581943 | 13q12.2 | PDX1 | New | 1.15 (1.10, 1.20) | 2.4x10^-9^ |
|  |  |  | rs16986825 | 22q12.1 | znrf3 | New | 1.18 (1.12, 1.25) | 1.2x10^-8^ |
|  |  |  | rs2736098 | 5p15.33 | exon2 of TERT | New | 0.80 (0.76, 0.85) | 9.78x10^-14^ |
|  |  |  | rs1561927 | 8q24.21 | PVT1 | Suggestive | 0.87 (0.83, 0.92) | 1.30x10^-7^ |
| Childs/2015^9^ | PanScan I, PanScan II, PanC4, PANDoRA: 9925 PDAC cases and 11,569 controls of European ancestry | 9925 | rs11655237 | 17q25.1 | LINC00673 | New | 1.26 (1.19, 1.34) | 1.42x10^-14^ |
|  |  |  | rs7214041 | 17q25.1 | LINC00673 | New | 1.26 (1.19, 1.34) | 2.88x10^-14^ |
|  |  |  | rs17688601 | 7p13 | SUGCT | New | 0.88 (0.84, 0.92) | 1.41x10^-8^ |
|  |  |  | rs9854771 | 3q29 | TP63 | New | 0.89 (0.85, 0.93) | 2.35x10^-8^ |
|  |  |  | rs1486134 | 2p13.3 | ETAA1 | Replicated | 1.14 (1.09, 1.19) | 3.36x10^-9^ |
| Zhang/2016^10^ | PanScan I-III: 5107 PDAC cases and 8845 controls of European ancestry | 5107 | rs2816938  rs10094872  rs35226131 | 1q32.1  8q24.21  5p15.33 | NR5A2  MYC  CLPTM1L-TERT | New  New  New | 1.20 (1.15, 1.25)  1.15 (1.10, 1.20)  0.71 (0.63, 0.80) | 4.88x10^-15^  3.22x10^-9^  1.70x10^-8^ |
| Klein/2018^11^ | PanScan I-III and PanC4: 9040 PDAC cases and 12,496 controls of European ancestry | 9040 | rs13303010  rs2941471  rs4795218  rs1517037 | 1p36.33  8q21.11  17q12  18q21.32 | NOC2L  HNF4G  HNF1B  GRP | New  New  New  New | 1.26 (1.19, 1.35)  0.89 (0.85, 0.93)  0.88 (0.84, 0.92)  0.86 (0.80, 0.91) | 8.36x10^-14^  6.60x10^-10^  1.32x10^-8^  3.28x10^-8^ |

# Supplementary Table 3. Study information of case-control studies of proteomics and PDAC

| **Reference** | **Study population** | **No. of cases** | **Platform** | **Identified biomarkers** | **Diagnostic performance** | **Validation** |
| --- | --- | --- | --- | --- | --- | --- |
| Wingren/2012/Sweden^12^ | PDAC (n=34), CP (n=16), AIP (n=23), HC (n=30) | 34 | Recombinant antibody microarray platform | A 25-serum biomarker signature discriminating PDAC from the combined group of NC, CP, and AIP | AUC: PDAC vs HC 0.95, PDAC vs CP 0.86, PDAC vs AIP 0.99 | Leave-one-out cross-validation: PDAC vs the entire group, AUC 0.88 |
| Faca/2008/US^13^ | 26 participants from the CARET cohort: PDAC cases with blood samples collected at least 1 year prior to a diagnosis (n=13) and matched-controls (n=13) | 13 | Proteomic approach based on extensive protein fractionation | 5 proteins that were up-regulated in mouse plasma at the PanIN stage (LCN2, REG1A, REG3, TIMP1, and IGFBP4) together with CA19.9 | AUC: 5 proteins 0.817, 5 proteins + CA 19-9 0.911 | NA |
| Ingvarsson/2008/Sweden^14^ | PDAC (n=24), HC (n=20) | 24 | Recombinant scFv antibody microarray | A protein signature based on 19 nonredundant analytes | A condensed set of biomarkers consisting of 19 nonredundant serum proteins differed significantly (*p*<0.05) between cancer and normal samples | The expression of four of the serum biomarkers in the signature, discriminating between PDAC and HC, were also confirmed using conventional ELISA (MCP-3, IL-4, IL-5, and IL-13) |
| Balasenthil/2011/US^15^ | PDAC (n=36), HC (n=19) | 36 | ELISA | Tissue factor pathway inhibitor (TFPI), tenascin C (TNC-FN III-C), CA 19-9 | AUC 0.99, sensitivity 90%, specificity 100%, or sensitivity 97.2%, specificity 90% | Balasenthil/2017/US^22^ |
| Brand/2011/US^16^ | PDAC (n=160), OPD (n=74), HC (n=107)  Validation: PDAC (n=173), OPD (n=70), HC (n=120) | 160 | The xMAP bead-based technology | CA 19-9, ICAM-1, OPG; CA 19-9, CEA, TIMP-1 | The panel of CA 19-9, ICAM-1, and OPG discriminated PDAC from HC with a sensitivity/specificity of 88/90% (AUC=0.93)  The panel of CA 19-9, CEA, and TIMP-1 discriminated PDAC from OPD subjects with a sensitivity/specificity of 76/90% (AUC=0.85) | The panel of CA 19-9, ICAM-1 and OPG discriminated PDAC from HC with a sensitivity/specificity of 78/94% (AUC=0.91)  The panel of CA19-9, CEA, and TIMP-1 discriminated PDAC from OPD a sensitivity/specificity of 71/89% (AUC=0.83) |
| Nie/2014/US^17^ | PDAC (n=37), pancreatic cyst (n=30), CP (n=30), HC (n=30) | 37 | LC-MS/MS analysis | 19 and 25 proteins were found to show significant differences in samples between PDAC and other conditions | 7 proteins were significantly different between PDAC cases and controls, which were further validated by ELISA and lectin-ELISA | The combination of α-1-antichymotrypsin (AACT), thrombospondin-1 (THBS1), and haptoglobin (HPT) outperformed CA 19-9 in distinguishing PDAC from HC (AUC 0.95 vs 0.89), diabetes (AUC 0.90 vs 0.85), cyst (AUC 0.82 vs 0.81), and CP (AUC 0.90 vs 0.79) |
| Gerdtsson/2015/Spain^18^ | PDAC (n=156), OPD (n=152), HC (n=30) | 156 | 293-plex recombinant antibody microarrays | PDAC vs others: IL-11, cytokines and chemokines (IL-11, IL-6, IL-13, IL-8, TNF-a, and Eotaxin), complement components (C1 inhibitor, C1q, C5, and Factor B), and enzymes (HADH2, GAK, and ATP-5B) | A multiplexed biomarker signature of up to 10 serum markers could discriminate PDAC from controls, with sensitivities and specificities in the 91-100% range (AUC=0.98)  PDAC vs OPD (AUC=0.70) | NA |
| Gerdtsson/2016/China^19^ | PDAC (n=118) by stage (resectable (I/II), locally advanced (III), and metastasis (IV)), and HC (n=95) | 118 | Recombinant antibody microarray platform | Properdin, vascular endothelial growth factor (VEGF), IL-8, complement factor (C3), calcineurin homologous protein-1 (CHP-1) | All PDAC stages could be discriminated from controls and the accuracy increased with disease progression, from stage I to IV (AUC): all PDAC vs HC 0.88, from stage I-IV 0.71, 0.86, 0.90, 0.93 | Leave-one-out cross-validation: all PDAC vs HC AUC 0.87 |
| Sogawa/2016/Japan^20^ | Pre- and postoperative sera of PDAC (n=3)  Validation set 1: 14 pre- and postoperative sera of PDAC  Validation set 2: HC (n=40), CP (n=20), PDAC (n=52)  Validation set 3: PDAC (n=66), stomach cancer (n=20), colorectal cancer (n=20), liver cancer (n=20), biliary tract cancer (n=20) | 80 | Tandem mass tag labelling and LC-MS/MS | C4b-binding protein a-chain (C4BPA) and polymeric immunoglobulin receptor (PIGR) | 20 proteins were selected whose serum levels were elevated more than two-fold before and after surgery in three pairs of sera from pre- and postoperative PDAC patients | PDAC vs HC and CP: AUC 0.860 for C4BPA, 0.846 for CA19-9, 0.930 for both  Early stage PDAC vs HC and CP: AUC 0.912 for C4BPA, 0.737 for CA 19.9, 0.868 for both  PDAC vs biliary tract cancer: AUC 0.854 for C4BPA, 0.264 for CA19-9 |
| Yoneyama/2016/Japan^21^ | IDACP (n=164), other gastrointestinal cancers (n=92), HC (n=106) | 164 | Antibody-based proteomics and LC-MS/MS-based proteomics | IGFBP2, IGFBP3 | HC vs IDACP: AUC for 23 proteins were greater than 0.80 | 38 stage I or II IDACP patients, and 65 HC: AUC 0.706 for IGFBP2 and 0.766 for IGFBP3, AUC 0.900 for the combination of IGFBP2, IGFBP3, and CA19-9  In the all-stage disease set: AUC 0.940 for the combination of IGFBP2, IGFBP3, and CA19-9 |
| Balasenthil/2017/US^22^ | Early-stage PDAC cohorts: stage I (n=43), stage II (n=163), CP (n=86), acute biliary obstruction (n=31), HC (n=108) | 206 | ELISA | TFPI, TNC-FN III-C, CA 19-9 | Validation of a functional genomics-based plasma migration signature biomarker panel | All early-stage vs HC: AUC 0.92, stage I/IIA 0.84, stage IIB 0.98, stage II 0.97  All early-stage vs CP: AUC 0.69-0.75  The panel improved CA 19.9 performance in all-early stage cohort (*p*<0.05) |
| Capello/2017/US^23^ | PDAC (n=187), benign pancreatic diseases (n=93), HC (n=169) | 187 | ELISA | TIMP1, LRG1, CA 19-9 | Validation of Faca et al.^47^ | Early-stage PDAC vs HC: sensitivity 63%, specificity 95%, AUC 0.95  Early-stage PDAC vs benign: sensitivity 35%, specificity 95%, AUC 0.85  The performance of the biomarker panel was statistically significantly improved compared with CA19-9 alone (*p*<0.001) |
| Liu/2017/China^24^ | HC (n=40), benign diseases (n=30, 4 pancreatitis, 13 pancreatic cyst, 13 benign tumour), PDAC (n=80) | 80 | Combined MS-intensive methods | Apolipoprotein E (ApoE), inter-alpha-trypsin inhibitor heavy chain H3 (ITIH3), apolipoprotein A-I (ApoA1), apolipoprotein L1 (ApoL1) | PDAC vs HC: 4 markers + CA 19-9: sensitivity 95%, specificity 94.1%, AUC 0.99; 4 markers: 85%, 94.1%, 0.94  PDAC vs HC and benign diseases: 4 markers + CA 19-9: 90%, 75%, 0.89; 4 markers: 85%, 80%, 0.87 | NA |
| Park/2017/Korea^25^ | Discovery set: HC (n=50), pancreatic benign diseases (n=34), PDAC (n=50)  Validation set: HC (N=349), pancreatic benign diseases (n=109), other cancer (n=149), PDAC (n=401) | 401 | LC-MS/MS | Leucine-rich alpha-2 glycopritein, transthyretin, CA 19-9 | Triplicate analysis to identity a 3-panel biomarker with sensitivity over 10% greater than that of CA 19-9 when specificity was fixed at 0.90 | PDAC vs HC: sensitivity 82.5%, specificity 92.1%, AUC 0.931  PDAC vs other cancer: sensitivity 82.5%, specificity 83.9%, AUC 0.899  PDAC vs pancreatic benign: sensitivity 82.5%, specificity 85.7%, AUC 0.892 |
| Park/2017/Korea^26^ | Discovery set: HC (n=182), pancreatitis (n=31), PDAC (n=116)  Validation set: HC (n=94), pancreatitis (n=70), PDAC (n=292) | 70 | SIS-MRM-MS | Apolipoprotein A-IV, apolipoprotein CIII, IGFBP2, tissue inhibitor of metalloproteinase 1 | The four proteins were significantly altered in PDAC cases in both the discovery and validation phase (*p*<0.01) | Early PDAC vs pancreatitis: a panel including CA 19-9, apolipoprotein A-IV, and tissue inhibitor of metalloproteinase 1 performed better than CA 19-9, AUC 0.934  PDAC vs HC: none of the four proteins alone was superior to CA 19-9 |
| Cohen/2017/US^27^ | Resectable PDAC (n=221) and HC (n=182) | 221 | The Bioplex 200 platform | ctDNA KRAS mutations and four proteins (CA 19-9, CEA, hepatocyte growth factor, osteoponin) | PDAC vs HC: sensitivity 64%, specificity 99.5% | NA |
| Cohen/2018/US^28^ | Early-stage PDAC (n=93, 89% stage II) and HC (n=812) | 93 | The Bioplex 200 platform | The presence of a mutation in 1933 distinct genomic positions or elevated levels of any of eight proteins (CA 125, CEA, CA 19-9, prolactin, hepatocyte growth factor, osteoponin, myeloperoxidase, TIMP-1) | PDAC vs HC: sensitivity 70%, specificity >99% | NA |

Abbreviation: AIP, autoimmune pancreatitis; AUC, area under the Receiver Operating Characteristic curve; CARET, Carotene and Retinol Efficacy Trial; CP, chronic pancreatitis; ELISA: enzyme-linked immunosorbent assays; HC, healthy control; IDACP, invasive ductal adenocarcinoma of pancreas; IGFBP, insulin-like growth factor-binding protein; iTRAQ, isobaric tags for relative and absolute quantification; LC-MS/MS, liquid chromatography-tandem mass spectrometry; OPD, other pancreas diseases; PDAC, pancreatic ductal adenocarcinoma; SIS-MRM-MS, stable isotope dilution-multiple reaction monitoring-mass spectrometry; WHI, Women's Health Initiative.

# Supplementary Table 4. Study information of case-control studies of metabolomics and PDAC

| **Reference** | **Study population** | **No. of cases** | **Platform** | **Identified biomarkers** | **Diagnostic performance** | **Validation** |
| --- | --- | --- | --- | --- | --- | --- |
| Bathe  /2011/Canada^29^ | PDAC (n=56), benign hepatobiliary disease (n=43) | 56 | ^1^H NMR | 3-hydro-xybutyrate, acetone | PDAC vs benign disease: AUC 0.837 | NA |
| Honda  /2012/Japan^30^ | Training: PDAC (n=112), sex- and age-matched HC (n=103)  Validation (n=833): 2 retrospective cohorts and a prospective cohort | 112 | oMALDI QqTOF- MS | ApoAII-2, ApoCIII-0 | PDAC vs HC:  ApoAII-2: AUC 0.877  ApoCIII-0: AUC 0.798 | PDAC vs HC:  AUC of ApoAII-2+CIII-0:  PDAC stage I-II 0.920  PDAC stage III-IV 0.918  All stages 0.919 |
| Zhang  /2012/China^31^ | PDAC (n=19), CP (n=20), HC (n=20) | 19 | ^1^H NMR | N-acetyl glycoprotein (NAG), VLDL, lipid glyceryl, dimethylamine (DMA), acetone, 3-hydroxybutyrate, citrate, lactate, LDL, HDL, valine, lysine, leucine, isoleucine, histidine, glutamine, glutamate, and alanine | PDAC vs HC: sensitivity 79%, specificity 85%  PDAC vs CP: sensitivity 84%, specificity 90% | NA |
| Kobayashi  /2013/Japan^32^ | Training: PDAC (n=43), HC (n=42)  Validation: PDAC (n=42), HC (n=41), CP (n=23) | 43 | GC-MS | Xylitol, 1,5-anhydro-D-glucitol, histidine, inositol | PDAC vs HC: sensitivity 86.0%, specificity 88.1%, AUC 0.93 | PDAC vs HC: sensitivity 71.4%, specificity 78.1%, AUC 0.76  False-positive rate in CP: 17.4% |
| Leichtle/  2013/Germany^33^ | PDAC (n=40), HC (n=40), CP (n=23) | 40 | Tandem MS | Amino acids by a neutral loss scan of 102 in the mass range of 130-280 or multiple reaction monitoring | Compared to CA 19-9 alone (AUC 0.53), the combined amino acid-based metabolite panel had a superior selectivity for the discrimination of HC, pancreatitis, and PDAC (AUC 0.89) | NA |
| Ritchie  /2013/Japan^34^ | Training: PDAC (n=40), HC (n=50)  Validation: PDAC (n=14), intraductal papillary mucinous neoplasms (IPMN, n=6), HC (n=40) | 40 | FI-FTICR-MS | 36-carbon ultralong-chain fatty acids, multiple choline-related systems (phosphatidylcholines, lyso-phosphatidylcholines, sphingomyelins, vinyl ether-containing plasmalogen ethanolamines) | PDAC vs HC:  AUC based on FI-MS/MS of selected markers from each system ranged between 0.93 and 0.97 | PDAC vs HC:  PC-594: AUC 0.97  IPMN vs HC: mean PC-594 significantly decreased (*p*=0.003) |
| Nicolardi/  2014/  Netherlands^35^ | Training: PDAC (n=49), HC (n=110)  Validation: PDAC (n=39), HC (n=75) | 49 | FTICR MS | Peptidome profiles all species up to 9kDa | Sensitivity and specificity >85%, AUC 0.92 | Sensitivity and specificity >85%, AUC 0.98 |
| Xie/2014/China, US^36^ | PDAC (n=200), HC (n=200) | 200 | GC/LC-MS | Glutamate, choline, 1,5-anhydro-D-glucitol, betaine, and methylguanidine | PDAC vs HC: sensitivity 97.7%, specificity 83.1%, AUC 0.943  PDAC stages 0-2: sensitivity 77.4% | PDAC vs HC: sensitivity 77.4%, specificity 75.8%, AUC 0.835  PDAC stages 0-2: sensitivity 84.8% |
| Di Gangi  /2015/Italy^37^ | PDAC (n=40) and HC (n=40) | 40 | Tandem MS | Palmitic acid, 1,2-dioleoylsn-glycero-3-phospho-rac-glycerol, lanosterol, lignoceric acid, 1-monooleoyl-rac-glycerol, cholesterol 5a,6a epoxide, erucic acid and taurolithocholic acid (T-LCA), oleoyl-L-carnitine, oleanolic acid | Palmitic acid (cut-off: 134.38 μM): AUC 1.00, sensitivity 100%, specificity 100% | NA |
| Fukutake/2015/Japan^38^ | PDAC (n=360), CP (n=28), HC (n=8372) | 360 | LC-MS | 6 plasma free amino acids: serine, asparagine, isoleucine, alanine, histidine, tryptophan | PDAC vs HC: AUC 0.89 | PDAC vs HC: 0.86, PDAC stage IIA to IIB vs HC: 0.81, PDAC vs CP: 0.87 |
| Ritchie  /2015/Canada^39^ | PDAC (n=84), HC (n=99) | 84 | Tandem MS | PC-594 fatty acid (a novel circulating 36-carbon long-chain polyunsaturated fatty acid) | PDAC-594>1.25 μmol/L:  RR 9.4 (5.0-17.7), AUC 0.93 (0.91-0.95) | NA |
| Akita/2016/Canada^40^ | PDAC (n=116), HC (n=138) | 116 | Triple quadrupole tandem MS | PC-594, lysophosphatidylcholine (lysoPC 18:2), phosphatidylcholine (18:0/18:2), sphingomyelin (18:1/24:0) | Validation of Ritchie et al.^48^ | PC-594: sensitivity 73.3%, specificity 92.0%, AUC 0.92  All four: sensitivity 86.2%, specificity 84.4% |
| Potjer/2016  /Netherlands^41^ | PDAC (n=5), HC (n=6) | 5 | FTICR MS | Serum peptides and proteins with isotopic resolution up to 15,000 Da | Cases had a higher median discriminant score than controls (*p*=0.001) | NA |
| He/2017/China^42^ | New-onset diabetes (n=30), patients with PDAC and new-onset diabetes (n=30) | 30 | LC-MS | N-succinyl-L-diaminopimelic-acid, phosphatidylethanolamine (18:2) | Sensitivity 93.3%, specificity 93.1%, AUC 0.95 | NA |
| Hirata/2017/Japan^43^ | Training set: early PDAC (n=55), HC (n=58)  Validation set: PDAC (n=16), HC (n=16) | 71 | GC-MS/tandem MS | Histidine, xylitol | Sensitivity 70.4%, specificity 89.5%, AUC 0.83  Adding CA19-9: sensitivity 90.7%, specificity 89.5%, AUC 0.93 | Sensitivity 75.0%, specificity 100.0%, AUC 0.89  Adding CA19-9: sensitivity 81.3%, specificity 93.8%, AUC 0.92 |
| Lindahl/2017/Sweden^44^ | Training set: PDAC (n=44) and CP (n=23)  Validation set: PDAC (n=20) and CP (n=31) | 44 | LC-MS | Glycocholic acid, N-palmitoyl glutamic acid, hexanoylcarnitine, phenylacetyl-glutamine, chenodeoxyglycocholate | OPLS-DA: R2X (cum) 0.39, Q2 (cum) 0.22, CV-ANOVA *p*=0.02 | OPLS-DA: R2X (cum) 0.74, Q2 (cum) 0.51, CV-ANOVA *p*=8.2x10^-7^ |
| Mehta/2017/US^45^ | PDAC (n=59), HC (n=48), CRC (n=66), T2DM (n=19) | 59 | SID-MRM based targeted MS | Lactate, lysoPC (18:2), alanine, choline, threonine, asparagine, tyrosine, lysine, palmitate, 3-hydroxybutyrate | PDAC vs HC: AUC 0.992  CRC vs HC: 0.986  T2DM vs HC: 0.957  CRC vs PDAC: 0.653  T2DM vs PDAC: 0.997 | NA |
| Mayerle  /2017  /Germany^46^ | PDAC (n=271), CP (n=282), liver cirrhosis (n=100), HC (n=261) | 271 | GC-MS, LC-MS/MS, solid-phase extraction-LC-MS/MS | 9 metabolites and CA19-9: proline, sphingomyelin (d18:2,C17:0), phosphatidycholine (C18:0,C22:6), isocitrate, sphinganine-1-phosphate (d18:0), histidine, pyruvate, ceramide (d18:1,C24:0), sphingomyelin (d17:1,C18:0) | PDAC vs CP:  Biomarker signature cut-off of 0.384 at 85% fixed specificity, sensitivity 94.9%, AUC 0.96 | PDAC vs CP:  AUC 0.94, sensitivity 89.9%, specificity 91.3%  PDAC vs HC:  AUC 0.90, sensitivity 89.9%, specificity 81.3% |

Abbreviations: ^1^H NMR, ^1^H nuclear magnetic resonance; ApoAII-2, hemi-truncated apolipoprotein AII dimer; ApoCIII-0, unglycosylated apolipoprotein CIII; AUC, area under the Receiver Operating Characteristic curve; CA 19-9, carbohydrate antigen 19-9; CP, chronic pancreatitis; CRC, colorectal cancer; CV-ANOVA, ANOVA of the cross-validated residuals; FTICR MS, fourier transform ion cyclotron resonance mass spectroscopy; GC-MS, gas chromatography mass spectrometry; HC, health control; LC-MS, liquid chromatography-mass spectroscopy; MS, mass spectroscopy; OPLS-MA, orthogonal partial least squares-discriminant analysis; oMALDI QqTOF MS, matrix-assisted laser desorption/ionization quadrupole time-of-flight mass spectrometry; PDAC, pancreatic ductal adenocarcinoma; RR, relative risk; SID-MRM, stable isotope labeling and multiple reaction monitoring; T2DM, type 2 diabetes.

# Supplementary Table 5. Case-control studies of metabolomics and PDAC reporting on phospholipids

| **Metabolite** | **Comparison** | **Estimate** | **Effect size** | **P-value** | **FDR p-value** |
| --- | --- | --- | --- | --- | --- |
| ***Mayerle/2017/Germany^45^*** |  |  |  |  |  |
| Sphingomyelin (d18:2,C17:0) | PDAC vs CP | Odds ratio | 1.15 | 1.19x10^-2^ | 0.07 |
| Phosphatidylcholine (C18:0,C22:6) |  |  | － | >0.05 | － |
| Sphingomyelin (d17:1,C18:0) |  |  | 1.37 | 4.61x10^-5^ | 0.008 |
| ***Mehta/2017/US^46^*** |  |  |  |  |  |
| LysoPC (18:2) | NC vs PDAC | Fold change | 0.49 | － | 1.45x10^-12^ |
|  | T2DM vs PDAC |  | 1.05 | － | 0.55 |
|  | CRC vs PDAC |  | 1.06 | － | 0.94 |
| ***He/2017/China^42^*** |  |  |  |  |  |
| Phosphatidylethanolamine (18:2) | PDAC vs new-onset DM | Odds ratio | 1.01 | 0.007 | － |

Abbreviations: CRC, colorectal cancer; DM, diabetes mellitus; FDR, false discovery rate; lysoPC, lysophosphatidylcholine; NC, normal control; PDAC, pancreatic ductal adenocarcinoma; PC, phosphatidylcholine.

# Supplementary Table 6. Prospective studies of blood lipids and risk of PDAC

| **Reference** | **No. of cases** | **Populations** | **Lipids** | **Results** |
| --- | --- | --- | --- | --- |
| Stolzenberg-Solomon/2002/Finland^49^ | 172 | The Alpha-Tocopherol, Beta-Carotene Cancer Prevention (ATBC) Study: 29,048 male smokers | TC, fasting | >276 vs <203 mg/dL: 1.13 (0.72, 1.76) |
|  |  |  | HDL-C, fasting | >55.2 vs <36 mg/dL: 1.05 (0.67, 1.65) |
| Ansary-Moghaddam/ 2006/ Asia-Pacific^50^ | 208 | The Asia Pacific Cohort Studies Collaboration: 420,310 Asian participants and 99,333 from Australia/New Zealand | TC, fasting | ≥5.8 vs <4.8 mmol/L: 0.88 (0.71, 1.10) |
| Berrington/2008/Korea^51^ | 2194 | The Korean Cancer Prevention Study: 1,213,829 enrolees in the National Health Insurance Corporation | TC, fasting | ≥240 vs <200 mg/dL: 0.97 (0.85, 1.10) |
| Batty/2009/UK^52^ | 163 | The Whitehall Study: 17898 men | TC, fasting | T3 vs T1: 0.71 (0.47, 1.07) Per 1.21mmol/L: 0.92 (0.78,1.08) |
| Inoue/2009/Japan^53^ | 65 | The Japan Public Health Center Study: 9548 men and 18,176 women | TG, random | High vs low (≥150mg/dL):  M 0.62 (0.23,1.63), F 1.23 (0.61, 2.49) |
|  |  |  | HDL-C, random | Low vs high (<40mg/dL M, <50mg/dL F):  M 0.59 (0.17, 2.06), F 1.52 (0.81, 2.86) |
| Meinhold/2009/Finland^54^ | 305 | The ATBC Study: 27,035 male smokers | HDL-C, fasting | Q5 vs Q1: 1.04 (0.72,1.50) |
| Johansen/2010/Europe^55^ | 862 | The Metabolic Syndrome and Cancer Project: 7 population-based cohorts in Austria, Norway, and Sweden, 577,315 participants | TC, random | Per 1-SD: M: 0.87 (0.79, 0.96), F: 1.09 (0.96, 1.22) |
|  |  |  | TG, random | Per 1-SD: M: 1.04 (0.94,1.15), F: 1.00 (0.88, 1.22) |
| Kuzmickiene/2013/Lithuania^56^ | 77 | 2 urban population-based cohorts in Lithuania with 7132 participants | TC, random | ≥6.63 vs <5.2 mmol/L: 1.76 (0.87, 3.55) |
| Wang/2015^57^ | 3677 | 6 cohort studies with 1,805,697 participants | TC, mixed | High vs low: 1.003 (0.859. 1.171) |
| Kabat/2018/US^58^ | 156 | 24,208 postmenopausal women in the Women's Health Initiative | TC, fasting | Q4 vs Q1:  0.76 (0.47, 1.21) |
|  |  |  | LDL-C, fasting | 0.92 (0.58, 1.46) |
|  |  |  | HDL-C, fasting | 0.53 (0.31, 0.89) |
|  |  |  | TG, fasting | 1.19 (0.72, 1.98) |

Abbreviation: F, female; HDL-C, high-density lipoprotein cholesterol; LDL-C, low-density lipoprotein cholesterol; M, male; TC, total cholesterol; TG, triglycerides.

References:

1. Zhou B, Xu JW, Cheng YG, Gao JY, Hu SY, Wang L, et al. Early detection of pancreatic cancer: Where are we now and where are we going? *Int J Cancer* 2017; 141(2): 231-41.

2. Zhang X, Shi S, Zhang B, Ni Q, Yu X, Xu J. Circulating biomarkers for early diagnosis of pancreatic cancer: facts and hopes. *Am J Cancer Res* 2018; 8(3): 332-53.

3. Archibugi L, Signoretti M, Capurso G. The microbiome and pancreatic cancer: an evidence-based association? *J Clin Gastroenterol* 2018; 52 (Suppl 1). doi: 10.1097/MCG.0000000000001092.

4. Imamura T, Komatsu S, Ichikawa D, Kawaguchi T, Miyamae M, Okajima W, et al. Liquid biopsy in patients with pancreatic cancer: Circulating tumor cells and cell-free nucleic acids. *World J Gastroenterol* 201; 22(25): 5627-41.

5. Low SK, Kuchiba A, Zembutsu H, Saito A, Takahashi A, Kubo M, et al. Genome-wide association study of pancreatic cancer in Japanese population. *PLoS One* 2010; 5(7): e11824.

6. Petersen GM, Amundadottir L, Fuchs CS, Kraft P, Stolzenberg-Solomon RZ, et al. A genome-wide association study identifies pancreatic cancer susceptibility loci on chromosomes 13q22.1, 1q32.1 and 5p15.33. *Nat Genet* 2010; 42(3): 224-8.

7. Wu C, Miao X, Huang L, Che X, Jiang G, Yu D, et al. Genome-wide association study identifies five loci associated with susceptibility to pancreatic cancer in Chinese populations. *Nat Genet* 2011; 44(1): 62-6.

8. Wolpin BM, Rizzato C, Kraft P, Kooperberg C, Petersen GM, Wang Z, et al. Genome-wide association study identifies multiple susceptibility loci for pancreatic cancer. *Nat Genet* 2014; 46(9): 994-1000.

9. Childs EJ, Mocci E, Campa D, Bracci PM, Gallinger S, Goggins M, et al. Common variation at 2p13.3, 3q29, 7p13 and 17q25.1 associated with susceptibility to pancreatic cancer. *Nat Genet* 2015; 47(8): 911-6.

10. Zhang M, Wang Z, Obazee O, Jia J, Childs EJ, Hoskins J, et al. Three new pancreatic cancer susceptibility signals identified on chromosomes 1q32.1, 5p15.33 and 8q24.21. *Oncotarget* 2016; 7(41): 66328.

11. Klein AP, Wolpin BM, Risch HA, Stolzenberg-Solomon RZ, Mocci E, Zhang M, et al. Genome-wide meta-analysis identifies five new susceptibility loci for pancreatic cancer. *Nat Commun* 2018; 9(1): 556.

12. Wingren C, Sandstrom A, Segersvard R, Carlsson A, Andersson R, [Lö](https://www.ncbi.nlm.nih.gov/pubmed/?term=L%C3%B6hr%20M%5BAuthor%5D&cauthor=true&cauthor_uid=22589272)hr M, et al. Identification of serum biomarker signatures associated with pancreatic cancer. *Cancer Res* 2012; 72(10): 2481-90.

13. Faca VM, Song KS, Wang H, Zhang Q, Krasnoselsky AL, Newcomb LF, et al. A mouse to human search for plasma proteome changes associated with pancreatic tumor development. *PLoS Med* 2008; 5(6): e123.

14. Ingvarsson J, Wingren C, Carlsson A, Ellmark P, Wahren B, Engström G, et al. Detection of pancreatic cancer using antibody microarray-based serum protein profiling. *Proteomics* 2008; 8(11): 2211-9.

15. Balasenthil S, Chen N, Lott ST, Chen J, Grizzle WE, Frazier ML, et al. A migration signature and plasma biomarker panel for pancreatic adenocarcinoma. *Cancer Prev Res* 2011; 4(1): 137-49.

16. Brand RE, Nolen BM, Zeh HJ, Allen PJ, Eloubeidi MA, Goldberg M, et al. Serum biomarker panels for the detection of pancreatic cancer. *Clin Cancer Res* 2011; 17(4): 805-16.

17. Nie S, Lo A, Wu J, Zhu J, Tan Z, Simeone DM, et al. Glycoprotein biomarker panel for pancreatic cancer discovered by quantitative proteomics analysis. *J Proteome Res* 2014; 13(4): 1873-84.

18. Gerdtsson AS, Malats N, Sall A, Real FX, Porta M, Skoog P, et al. A multicenter trial defining a serum protein signature associated with pancreatic ductal adenocarcinoma. *Int J Proteomics* 2015; 2015: 587250.

19. Gerdtsson AS, Wingren C, Persson H, Delfani P, Nordström M, Ren H, et al. Plasma protein profiling in a stage defined pancreatic cancer cohort - Implications for early diagnosis. *Mol Oncol* 2016; 10(8): 1305-16.

20. Sogawa K, Takano S, Iida F, Satoh M, Tsuchida S, Kawashima Y, et al. Identification of a novel serum biomarker for pancreatic cancer, C4b-binding protein alpha-chain (C4BPA) by quantitative proteomic analysis using tandem mass tags. *Br J Cancer* 2016; 115(8): 949-56.

21. Yoneyama T, Ohtsuki S, Honda K, Kaboyashi M, Iwasaki M, Uchida Y, et al. Identification of IGFBP2 and IGFBP3 as compensatory biomarkers for CA19-9 in early-stage pancreatic cancer using a combination of antibody-based and LC-MS/MS-based proteomics. *PLoS One* 2016; 11(8): e0161009.

22. Balasenthil S, Huang Y, Liu S, Marsh T, Chen J, Stass SA, et al. A plasma biomarker panel to identify surgically resectable early-stage pancreatic cancer. *J Natl Cancer Inst* 2017; 109(8).

23. Capello M, Bantis LE, Scelo G, Zhao Y, Li P, Dhillon DS, et al. Sequential validation of blood-based protein biomarker candidates for early-stage pancreatic cancer. *J Natl Cancer Inst* 2017; 109(4).

24. Liu X, Zheng W, Wang W, Shen H, Liu L, Lou W, et al. A new panel of pancreatic cancer biomarkers discovered using a mass spectrometry-based pipeline. *Br J Cancer* 2017; 117(12): 1846.

25. Park J, Choi Y, Namkung J, Yi SG, Kim H, Yu J, et al. Diagnostic performance enhancement of pancreatic cancer using proteomic multimarker panel. *Oncotarget* 2017; 8(54): 93117.

26. Park J, Lee E, Park KJ, Park HD, Kim JW, Woo HI, et al. Large-scale clinical validation of biomarkers for pancreatic cancer using a mass spectrometry-based proteomics approach. *Oncotarget* 2017; 8(26): 42761.

27. Cohen JD, Javed AA, Thoburn C, Wong F, Tie J, Gibbs P, et al. Combined circulating tumor DNA and protein biomarker-based liquid biopsy for the earlier detection of pancreatic cancers. *Proc Natl Acad Sci U S A* 2017; 114(38): 10202-7.

28. Cohen JD, Li L, Wang Y, Thoburn C, Afsari B, Danilova L, et al. Detection and localization of surgically resectable cancers with a multi-analyte blood test. *Science* 2018; 359(6378): 926-30.

29. Bathe OF, Shaykhutdinov R, Kopciuk K, Weljie AM, McKay A, Sutherland FR, et al. Feasibility of identifying pancreatic cancer based on serum metabolomics. *Cancer Epidemiol Biomarkers Prev* 2011; 20(1): 140-7.

30. Honda K, Okusaka T, Felix K, Nakamori S, Sata N, Nagai H, et al. Altered plasma apolipoprotein modifications in patients with pancreatic cancer: protein characterization and multi-institutional validation. *PLoS One* 2012; 7(10): e46908.

31. Zhang L, Jin H, Guo X, Yang Z, Zhao L, Tang S, et al. Distinguishing pancreatic cancer from chronic pancreatitis and healthy individuals by (1)H nuclear magnetic resonance-based metabonomic profiles. *Clin Biochem* 2012; 45(13-14): 1064-9.

32. Kobayashi T, Nishiumi S, Ikeda A, Yoshie T, Sakai A, Matsubara A, et al. A novel serum metabolomics-based diagnostic approach to pancreatic cancer. *Cancer Epidemiol Biomarkers Prev* 2013; 22(4): 571-9.

33. Leichtle AB, Ceglarek U, Weinert P, Nakas CT, Nuoffer JM, Kase J, et al. Pancreatic carcinoma, pancreatitis, and healthy controls: metabolite models in a three-class diagnostic dilemma. *Metabolomics* 2013; 9(3): 677-87.

34. Ritchie SA, Akita H, Takemasa I, Equchi H, Pastural E, Nagano H, et al. Metabolic system alterations in pancreatic cancer patient serum: potential for early detection. *BMC Cancer* 2013; 13: 416.

35. Nicolardi S, Velstra B, Mertens BJ, Bonsing B, Mesker WE, Tollenaar R, et al. Ultrahigh resolution profiles lead to more detailed serum peptidome signatures of pancreatic cancer. *Advances in Integrative Medicine* 2014; 2: 39-51.

36. Xie G, Lu L, Qiu Y, Ni Q, Zhang W, Gao YT, et al. Plasma metabolite biomarkers for the detection of pancreatic cancer. *J Proteome Res* 2014; 14(2): 1195-202.

37. Di Gangi IM, Mazza T, Fontana A, Copetti M, Fusilli C, Ippolito A, et al. Metabolomic profile in pancreatic cancer patients: a consensus-based approach to identify highly discriminating metabolites. *Oncotarget* 2016; 7(5): 5815-29.

38. Fukutake N, Ueno M, Hiraoka N, Shimada K, Shiraishi K, Saruki N, et al. A novel multivariate index for pancreatic cancer detection based on the plasma free amino acid profile. *PLoS One* 2015; 10(7): e0132223.

39. Ritchie SA, Chitou B, Zheng Q, Jayasinghe D, Jin W, Mochizuki A, et al. Pancreatic cancer serum biomarker PC-594: Diagnostic performance and comparison to CA19-9. *World J Gastroenterol* 2015; 21(21): 6604-12.

40. Akita H, Ritchie SA, Takemasa I, Equchi H, Pastural E, Jin W, et al. Serum metabolite profiling for the detection of pancreatic cancer: results of a large independent validation study. *Pancreas* 2016; 45(10): 1418-23.

41. Potjer TP, Mertens BJ, Nicolardi S, van der Burgt YE, Bonsing BA, Mesker WE, et al. Application of a serum protein signature for pancreatic cancer to separate cases from controls in a pancreatic surveillance cohort. *Transl Oncol* 2016; 9(3): 242-7.

42. He X, Zhong J, Wang S, Zhou Y, Wang L, Zhang Y, et al. Serum metabolomics differentiating pancreatic cancer from new-onset diabetes. *Oncotarget* 2017; 8(17): 29116.

43. Hirata Y, Kobayashi T, Nishiumi S, Yamanaka K, Nakagawa T, Fujigaki S, et al. Identification of highly sensitive biomarkers that can aid the early detection of pancreatic cancer using GC/MS/MS-based targeted metabolomics. *Clinica Chimica Acta* 2017; 468: 98-104.

44. Lindahl A, Heuchel R, Forshed J, Lehtiö J, Löhr M, Nordström A. Discrimination of pancreatic cancer and pancreatitis by LC-MS metabolomics. *Metabolomics* 2017; 13(5): 61.

45. Mehta KY, Wu H-J, Menon SS, Fallah Y, Zhong X, Rizk N, et al. Metabolomic biomarkers of pancreatic cancer: a meta-analysis study. *Oncotarget* 2017; 8(40): 68899.

46. Mayerle J, Kalthoff H, Reszka R, Kamlage B, Peter E, Schniewind B, et al. Metabolic biomarker signature to differentiate pancreatic ductal adenocarcinoma from chronic pancreatitis. *Gut* 2018; 67(1): 128-37.

47. Faca VM, Song KS, Wang H, Zhang Q, Krasnoselsky AL, Newcomb LF, et al. A mouse to human search for plasma proteome changes associated with pancreatic tumor development. *PLoS Med* 2008; 5(6): e123.

48. Risch HA, Yu H, Lu L, Kidd MS. Detectable symptomatology preceding the diagnosis of pancreatic cancer and absolute risk of pancreatic cancer diagnosis. *Am J Epidemiol* 2015; 182(1): 26-34.

49. Stolzenberg-Solomon RZ, Pietinen P, Taylor PR, Virtamo J, Albanes D. A prospective study of medical conditions, anthropometry, physical activity, and pancreatic cancer in male smokers. *Cancer Causes Control* 2002; 13(5): 417-26.

50. Ansary-Moghaddam A, Huxley R, Barzi F, Lawes C, Ohkubo T, Fang X, et al. The effect of modifiable risk factors on pancreatic cancer mortality in populations of the Asia-Pacific region. *Cancer Epidemiol Biomarkers Prev* 2006; 15(12): 2435-40.

51. Berrington de Gonzalez A, Yun JE, Lee SY, Klein AP, Jee SH. Pancreatic cancer and factors associated with the insulin resistance syndrome in the Korean cancer prevention study. *Cancer Epidemiol Biomarkers Prev* 2008; 17(2): 359-64.

52. Batty GD, Kivimaki M, Morrison D, Huxley R, Smith GD, Clarke R, et al. Risk factors for pancreatic cancer mortality: extended follow-up of the original Whitehall Study. *Cancer Epidemiol Biomarkers Prev* 2009; 18(2): 673-5.

53. Inoue M, Noda M, Kurahashi N, Iwasaki M, Sasazuki S, Iso H, et al. Impact of metabolic factors on subsequent cancer risk: results from a large-scale population-based cohort study in Japan. *Eur J Cancer Prev* 2009; 18(3): 240-7.

54. Meinhold CL, Berrington de Gonzalez A, Albanes D, Weinstein SJ, Taylor PR, Virtamo J, et al. Predictors of fasting serum insulin and glucose and the risk of pancreatic cancer in smokers. *Cancer Causes Control* 2009; 20(5): 681-90.

55. Johansen D, Stocks T, Jonsson H, Lindkvist B, Björge T, Concin H, et al. Metabolic factors and the risk of pancreatic cancer: a prospective analysis of almost 580,000 men and women in the Metabolic Syndrome and Cancer Project. *Cancer Epidemiol Biomarkers Prev* 2010; 19(9):2307-17.

56. Kuzmickiene I, Everatt R, Virviciute D, Tamosiunas A, Radisauskas R, Reklaitiene R, et al. Smoking and other risk factors for pancreatic cancer: A cohort study in men in Lithuania. *Cancer Epidemiol* 2013; 37(2): 133-9.

57. Wang J, Wang WJ, Zhai L, Zhang DF. Association of cholesterol with risk of pancreatic cancer: a meta-analysis. *World J Gastroenterol* 2015; 21(12): 3711-9.

58. Kabat GC, Kim MY, Chlekowski RT, Vitolins MZ, Wassertheil-Smoller S, Rohan TE. Serum lipids and risk of obesity-related cancers in postmenopausal women. *Cancer Causes Control* 2018; 29(1): 13-24.
